# Coordination of Immune-Stroma Crosstalk by IL-6 Family Cytokines

**DOI:** 10.3389/fimmu.2019.01093

**Published:** 2019-05-15

**Authors:** Nathaniel R. West

**Affiliations:** Department of Cancer Immunology, Genentech, South San Francisco, CA, United States

**Keywords:** stromal cells, cytokines, inflammation, fibrosis, immune

## Abstract

Stromal cells are a subject of rapidly growing immunological interest based on their ability to influence virtually all aspects of innate and adaptive immunity. Present in every bodily tissue, stromal cells complement the functions of classical immune cells by sensing pathogens and tissue damage, coordinating leukocyte recruitment and function, and promoting immune response resolution and tissue repair. These diverse roles come with a price: like classical immune cells, inappropriate stromal cell behavior can lead to various forms of pathology, including inflammatory disease, tissue fibrosis, and cancer. An important immunological function of stromal cells is to act as information relays, responding to leukocyte-derived signals and instructing leukocyte behavior in kind. In this regard, several members of the interleukin-6 (IL-6) cytokine family, including IL-6, IL-11, oncostatin M (OSM), and leukemia inhibitory factor (LIF), have gained recognition as factors that mediate crosstalk between stromal and immune cells, with diverse roles in numerous inflammatory and homeostatic processes. This review summarizes our current understanding of how IL-6 family cytokines control stromal-immune crosstalk in health and disease, and how these interactions can be leveraged for clinical benefit.

## The Diverse Roles of Stromal Cells in Immunity and Inflammation

The term “stroma” refers to the non-parenchymal components of tissues that form a supportive matrix in which parenchymal cells reside (1). While a confusingly broad array of cell types have been described as “stromal cells,” in this review they are defined as non-hematopoietic, non-epithelial mesenchymal cells, including fibroblasts, myofibroblasts, bone marrow stromal cells, and the specialized fibroblast-like stromal cells of secondary lymphoid organs. Other mesenchymal populations such as endothelial cells, adipocytes, and muscle cells, while of great interest, are largely omitted from this discussion for the sake of brevity and clarity. Long considered to be mere structural entities without specialized functions, an explosion of data in the last two decades has established stromal cells as key regulators of both protective and pathological immune responses (2).

Regulation of immune function by stromal cells has been most extensively studied in the context of secondary lymphoid organs. First identified in 1992, podoplanin (PDPN)^+^ fibroblastic reticular cells (FRC) form a dense reticular network in lymph nodes that facilitates leukocyte migration and antigen presentation (2–5). By producing soluble chemokines, cytokines, and other factors—such as CCL19 (C-C motif chemokine ligand 19), CCL21, and IL-7 (interleukin 7)—FRC are crucial for controlling leukocyte recruitment, survival, and proliferation. FRC-like stromal cells play similar roles in other lymphatic tissues, such as in tertiary lymphoid organs of the intestinal mucosa (6, 7). In non-lymphoid tissues, stromal cells can exert similar effects to those of the secondary lymphoid organs by acting as scaffolds for leukocyte migration and by producing a diverse array of cytokines and chemokines (2).

Importantly, the immunological functions of stromal cells can vary substantially depending on their host organ and physiological context. For example, lymph node FRC recruit CCR7 (C-C chemokine receptor type 7)^+^ T cells (naïve and central memory) and CCR7^+^ dendritic cells (DC) to lymph nodes by producing the chemokines CCL19 and CCL21, as well as the pro-survival cytokines IL-7 and IL-15, thereby coordinating T cell activation and maintenance (4). In contrast, stromal cells in peripheral tissues generally lack expression of CCL19 and CCL21; accordingly, naïve and central memory T cells are infrequent in the periphery. However, expression of various pattern recognition and cytokine receptors by non-lymphoid tissue stromal cells allows them to sense microbial molecules and endogenous danger signals (1, 8, 9). In response, they produce chemokines [including CCL20 and CXCL10 (C-X-C motif chemokine ligand 10)] that attract effector T cells to sites of inflammation. Furthermore, inducible expression of leukocyte adhesion molecules including ICAM-1 (intercellular adhesion molecule 1) and VCAM-1 (vascular cell adhesion molecule 1) allows tissue-resident stromal cells to further influence the balance between leukocyte recruitment, retention, and recirculation (1, 2, 9). Finally, stromal cells contribute directly to immune response resolution and tissue repair, the latter being one of their best studied functions. Examples of “pro-resolution” factors produced by stromal cells include NOS2 (nitric oxide synthase 2) and NO (nitric oxide), which are released by lymph node FRC to constrain T cell proliferation (10–12), and IDO1 (indoleamine 2,3-dioxygenase 1) produced by peripheral stromal cells, which similarly limits T cell proliferation by depleting the critical T cell metabolite tryptophan (13, 14). Thus, stromal cells in different tissues collectively regulate the strength, quality, and duration of immune responses via diverse and complementary mechanisms.

As with most immunological processes, communication between stromal and immune cells is highly dependent on cytokines. Stromal cells bear receptors to a variety of biologically diverse cytokines that represent virtually all branches of innate and adaptive immunity, including innate inflammatory cytokines [e.g., TNF (tumor necrosis factor) and IL-1β], Th1 cytokines [e.g., IFN-γ (interferon gamma)], Th2 cytokines (e.g., IL-13), Th17 cytokines (e.g., IL-17A), and tolerogenic cytokines [e.g., TGF-β (transforming growth factor beta)] (7, 9, 15, 16). In turn, stromal cells can be prodigious producers of other cytokines and chemokines, such as IL-6 (1, 2, 7, 9). In recent years, cytokines of the IL-6 family have gained increasing attention for their roles in various homeostatic and pathological processes, which in many cases can be attributed to their ability to co-ordinate immune-stroma crosstalk. This review aims to provide a focused update on the contributions of IL-6 family members to immune-stromal interactions.

## An Overview of the IL-6 Cytokine Family

**Figure 1:**
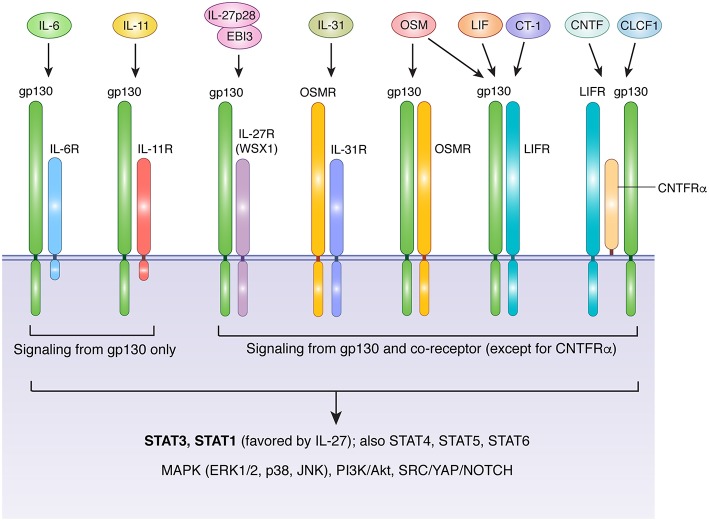
Receptor usage of IL-6 family cytokines. With the exception of IL-31, IL-6 family cytokines transduce signals via receptor complexes that include gp130 and one or more additional ligand-specific subunits. IL-6 and IL-11 signaling requires IL-6R and IL-11R, respectively. The cytoplasmic domains of these receptor are short and lack signaling motifs, making gp130 the sole source of signal transduction downstream of IL-6 and IL-11. The heterodimeric cytokine IL-27 (comprised of IL-27p28 and EBI3) requires a complex of gp130 and IL-27RA. LIF and CT-1 use a heterodimeric complex of gp130 and LIFR, while CNTF and CLCF1 signal via a trimeric complex of gp130, LIFR, and CNTFRα, a GPI-anchored protein that does not directly contribute to signaling beyond facilitation of ligand binding. OSM displays species-specific receptor usage. In humans and rats, OSM signals via either gp130/OSMR or gp130/LIFR complexes, while in mice OSM is primarily recognized by OSMR. IL-31 does not require gp130, and instead uses a complex of OSMR and IL-31R. Aside from IL-6R, IL-11R, and CNTFRα, all receptors in the IL-6 family are capable of directly activating signal transduction in response to ligand binding. IL-6 family cytokines employ classical JAK-mediated signaling. Major downstream mediators include STAT3 (the main STAT for all except IL-27), STAT1 (activated preferentially by IL-27 and to a lesser extent by other IL-6 family members), additional STATs that depend on cell type and physiological context (including STATs 4, 5, and 6), the MAPK cascade, PI3K/Akt/mTOR signaling, and SRC/YAP/NOTCH signaling. Akt, protein kinase B; CLCF1, cardiotrophin-like cytokine factor 1; CNTF, ciliary neurotrophic factor; CT-1, cardiotrophin 1; EBI3, Epstein-Barr virus induced 3; ERK, extracellular signal-regulated kinase; gp130, glycoprotein 130, also known as IL-6 signal transducer; IL, interleukin; IL-6R, IL-6 receptor; IL-11R, IL-11 receptor; IL-27RA, IL-27 receptor; CNTFRα, CNTF receptor; LIF, leukemia inhibitory factor; LIFR, LIF receptor; MAPK, mitogen activated protein kinase; JAK, janus kinase; JNK, c-jun n-terminal kinase; mTOR, mammalian target of rapamycin; OSM, oncostatin M; OSMR, OSM receptor; PI3K, phosphatidylinositol-3-kinase; STAT, signal transducer and activator of transcription; SRC, Proto-oncogene tyrosine-protein kinase Src; YAP, yes-associated protein.

The IL-6 family includes IL-6, IL-11, IL-27, IL-31, oncostatin M (OSM), leukemia inhibitory factor (LIF), ciliary neurotrophic factor (CNTF), cardiotrophin 1 (CT-1), and cardiotrophin-like cytokine factor 1 (CLCF1) (17, 18). With the exception of IL-27, which is a heterodimeric protein comprised of IL-27p28 and EBI3 (Epstein-Barr virus-induced gene 3) (19), IL-6 family members are compact 4-helix bundle cytokines made from a single polypeptide. Glycoprotein 130 (gp130, encoded by the *IL6ST* gene) is a crucial receptor subunit utilized by all members of the IL-6 family except IL-31. While gp130 expression is relatively ubiquitous in a wide variety of tissues and organs, cell-type specificity for different IL-6 family members is bestowed by the more restricted expression patterns of ligand-specific co-receptors, including IL-6R (IL-6 receptor), IL-11R (IL-11 receptor), IL-27Rα (IL-27 receptor alpha), OSMR (OSM receptor), LIFR (LIF receptor), and CNTFRα (CNTF receptor alpha). Three distinct forms of receptor-ligand complexes have been described (Figure 1). First characterized was that of IL-6, which engages IL-6R along with two subunits of gp130. Intriguingly, although this implies the formation of a trimeric complex, a series of cooperative interactions can ultimately produce an interlocked hexamer comprised of two subunits each of IL-6, IL-6R, and gp130 (20). A similar structure is likely formed in response to IL-11/IL-11R interaction (21, 22). In this arrangement, only gp130 drives signal transduction, due to an absence of intracellular signaling motifs in IL-6R and IL-11R. In contrast, OSMR, LIFR, and IL-27Rα form heterodimers with gp130 in the presence of their cognate ligands (23–28). Unlike IL-6R and IL-11R, OSMR, LIFR, and IL-27Rα are capable of driving signal transduction via their own suite of signaling motifs. Finally, CNTF and CLCF1 drive formation of a trimeric complex that includes gp130, LIFR, and CNTFRα (29–31). The gp130-independent outlier of the family, IL-31, engages a heterodimeric complex of IL-31Rα (previously known as gp130-like receptor) and OSMR (18). Notably, while mouse OSM binds with high affinity only to the gp130/OSMR heterodimer, human and rat OSM can bind with high affinity to either gp130/OSMR or gp130/LIFR heterodimers (32–34). Thus, in rats and humans, manipulation of LIFR would be expected to affect both OSM and LIF signaling (as well as CLCF1, CT-1, and CNTF), while manipulation of OSMR would influence OSM and IL-31 signaling. As a corollary, changes in human or rat OSM bioavailability would influence cells that express OSMR and/or LIFR, while changes in LIF or IL-31 would affect only LIFR- or IL-31Rα-expressing cells, respectively.

All members of the IL-6 family drive signal transduction via receptor-associated Janus kinases (primarily JAK1 and JAK2), which phosphorylate a variety of conserved tyrosine residues in the cytoplasmic domains of signaling receptor subunits (gp130, OSMR, LIFR, IL-27Rα, and IL-31Rα) (17, 18, 35). Several downstream signaling pathways are activated in response, including signal transducer and activator of transcription (STAT) proteins (including STAT1, STAT3, STAT4, STAT5, and STAT6), the mitogen-activated protein kinase (MAPK) cascade, the phosphatidylinositol-3-kinase (PI3K)/Akt pathway, and the SRC/YAP/NOTCH pathway (Figure 1). While signal transduction by individual IL-6 family members is broadly similar, the relative strength of activation of specific pathways can differ depending on the cytokine, cell type, and physiological context. For example, unlike gp130, OSMR efficiently recruits the adapter protein SHC, allowing OSM to drive more potent activation of the MAPK pathway than IL-6, which signals via SHP-2 bound to gp130 (35, 36). Similarly, although STAT3 is generally considered to be the dominant STAT protein activated by the IL-6 family, IL-27 preferentially activates STAT1 (37). Further complexity is provided by the capacity of IL-6, IL-11, and CNTF to signal via soluble receptor forms in a process known as *trans* signaling. In this process, soluble versions of IL-6R, IL-11R, or CNTFRα are produced either through proteolytic cleavage of membrane-bound receptors, or via expression of alternatively spliced mRNA; in either case, the soluble receptor form can dimerize with its cognate ligand in solution, and subsequently produce a functional signaling complex in association with membrane-bound gp130 (18, 38–40). Cells thus require only gp130 to be sensitive to *trans* signaling, which allows many cell types that lack IL-6R, IL-11R, or CNTFRα to respond to these cytokines. In the case of IL-6, *trans* signaling is thought to be a critical mechanism by which IL-6 promotes disease pathogenesis, particularly arthritis and colorectal cancer (18, 41, 42). Thus, while many similarities exist between IL-6 family cytokines, differences in their receptor usage, signal transduction profiles, and patterns of receptor expression collectively foster a substantial degree of functional pleiotropy. Indeed, IL-6 family members influence cell survival, proliferation, differentiation, metabolism, and migration, thus contributing to a plethora of physiological processes that are critical for both homeostasis and pathology.

## Expression of IL-6 Family Cytokines by Stromal Cells

**Figure 2:**
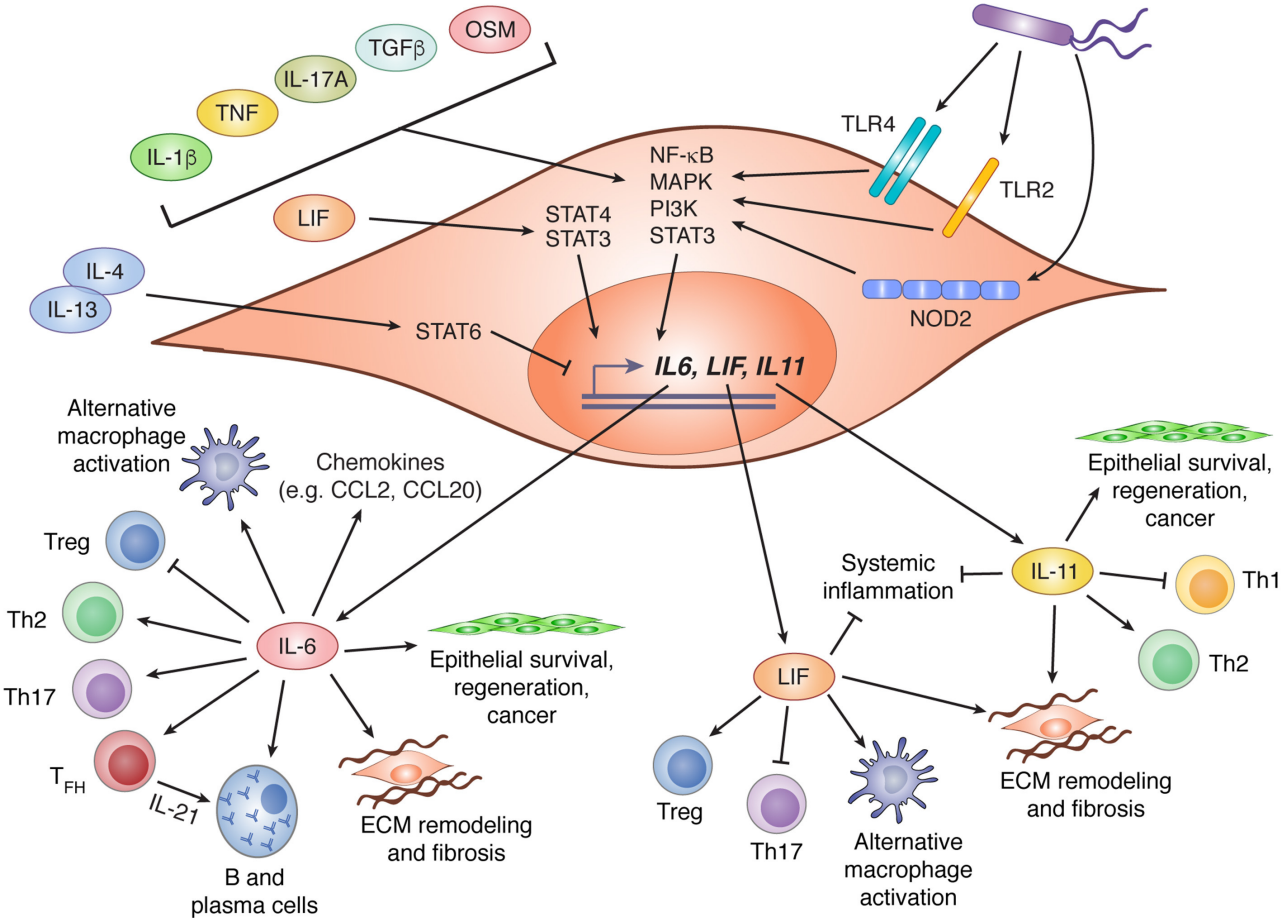
IL-6 family cytokine production by stromal cells and their biological effects. Stromal cells are important contributors to production of three members of the IL-6 family: IL-6, LIF, and IL-11. Expression of these cytokines is regulated by various stimuli including recognition of bacterial products via TLR2, TLR4, or NOD2, and diverse cytokines that drive activation of NF-κB, MAPK, PI3K, and STAT3. LIF has been shown to promote IL-6 expression via STAT4 signaling, while IL-4 and IL-13 can suppress LIF and IL-11 expression through activation of STAT6. Following production by stromal cells, IL-6, LIF, and IL-11 can influence diverse biological processes including CD4^+^ T cell polarization, regulation of chemokine production, promotion of alternative macrophage differentiation, and tissue remodeling through effects on stromal and epithelial cells. In this figure, arrows indicate stimulatory effects, and capped lines indicate inhibitory effects. All processes illustrated are described further in the main text. CCL, C-C motif chemokine ligand; ECM, extracellular matrix; IL, interleukin; LIF, leukemia inhibitor factor; MAPK, mitogen activated protein kinase; NF-κB, nuclear factor kappa B; NOD2, nucleotide-binding oligomerization domain-containing protein 2; OSM, oncostatin M; PI3K, phosphatidylinositol-3-kinase; STAT, signal transducer and activator of transcription; T_FH_, T follicular helper cell; TGFβ, transforming growth factor beta; Th, T helper; TLR, toll-like receptor; Treg, regulatory T cell.

Although some members of the IL-6 family are produced primarily by hematopoietic cells (notably OSM and IL-27), stromal cells can be important sources of several others, including IL-6, IL-11, and LIF. Diverse factors appear to regulate the expression of these cytokines by stromal cells, including microbial sensing, detection of endogenous alarmins, stimulation by other cytokines (including those within the IL-6 family itself), and cell stress (Figure 2). Although these inputs are known drivers of cytokine production, the critical drivers *in vivo*, particularly under physiological conditions, are rarely well defined.

In response to infection or an inflammatory challenge, IL-6 production is rapidly increased by stromal cells. Depending on their location and the nature of the challenge, this could be due to direct sensing of danger signals, responses to other inflammatory cytokines, or both. As an NF-κB (nuclear factor kappa B) response gene (43), IL-6 is induced by stromal cells downstream of several pattern recognition receptors including, but probably not limited to, toll-like receptor (TLR)2, TLR4, and NOD2 (nucleotide binding oligomerization domain 2) (44–46). The NF-κB activating cytokines IL-1β, IL-17A, and TNF (tumor necrosis factor alpha) are also potent inducers of stromal IL-6 production, and can do so in synergy with one another (43, 47–54). Although NF-κB is thought to be the dominant driver of IL-6 production downstream of these cytokines, contributions by MAPK signaling have also been observed. Indeed, signaling by alternative pathways such as the MAPK and PI3K cascades may underlie the ability of cytokines like OSM (55, 56), IL-4 (49), and TGF-β (54, 57) to promote stromal IL-6 expression, since these are not classical activators of NF-κB. Beyond cytokines and danger signals, cadherin-11 (CDH11), a mesenchymal cadherin that engages in homophilic interactions between adjacent cells, has also been shown to drive IL-6 production via NF-κB and MAPK signaling (53). Indeed, blockade of CDH11 attenuates inflammation in mouse models of arthritis, an effect that may be due in part to reduced IL-6 production by CDH11^+^ synovial fibroblasts (53). Finally, IL-6 is a well-known product of the senescence-associated secretory phenotype (SASP) in fibroblasts, a feature associated with aging and cancer (58). Indeed, IL-6 produced by prostate tumor fibroblasts in response to metabolic stress has been proposed to mediate malignant progression (59).

Less is known about the regulation of LIF and IL-11 expression by stromal cells, but the mechanisms involved may be similar to those of IL-6. Like IL-6, LIF and IL-11 expression by stromal cells can be induced by IL-1β, TNF, and TGF-β (60–64). Notably, induction of both IL-11 and LIF in response to TGF-β stimulation of cancer-associated fibroblasts is thought to promote tumor progression (61, 62). Intriguingly, IL-4 and IL-13 were shown to counteract TNF and IL-1β-induced expression of LIF and IL-11, but not IL-6, by gingival fibroblasts (64). This effect was dependent on STAT6, and provides a potential mechanism for selective modulation of individual IL-6 family members.

## Responsiveness of Stromal Cells to IL-6 Family Cytokines

Stromal cells express the necessary receptor subunits to respond to the majority of gp130-dependent IL-6 family cytokines. In general, gp130 and OSMR are ubiquitously expressed by stromal cells from essentially all anatomical locations studied thus far. OSM is therefore a major activating factor of stromal cells, as well as various other mesenchymal populations including endothelial cells, muscle cells, adipocytes, and osteoblasts (34, 56, 65). Expression of other ligand-specific receptor subunits is more variable and depends on the cell type, anatomical location, and physiological context. IL-6R, for example, tends to be expressed at relatively low levels, and stromal cells are correspondingly less sensitive to classical IL-6 signaling than OSM. Indeed, expression of OSMR mRNA in human colon fibroblasts is roughly 10x higher than that of IL-6R (55). However, inflammatory conditions that yield soluble IL-6R can increase stromal cell sensitivity to IL-6 due to *trans* signaling. Responsiveness of stromal cells to LIF appears to vary widely depending on anatomical location. For example, LIF induces contractile and inflammatory phenotypes in dermal and synovial fibroblasts, respectively, but has little effect on colon fibroblasts (55, 62, 63, 66). Sensitivity of stromal cells to IL-11 and IL-27 has also been documented (67–73). In contrast, IL-31Rα does not seem to be expressed by most stromal cells at physiologically relevant levels (74, 75).

## Control of Inflammation and Adaptive Immunity by the IL-6-Stroma Axis

Exposure of stromal cells to factors such as microbial ligands or inflammatory cytokines can drive IL-6 production during both acute and chronic inflammation. Following infection of mice by *Toxoplasma gondii*, for example, IL-6 expression was shown to be elevated in a population of bone marrow stromal cells characterized by high VCAM-1 and low CD146 expression, and stroma-derived IL-6 was required for the increased myelopoiesis that occurs as part of the host response to infection (76). Bone marrow stromal cells also induce IL-6 in response to viral infections such as CMV (cytomegalovirus) (77). During *Helicobacter hepaticus*-driven colitis in mice, non-hematopoietic stromal cells are the dominant intestinal producers of IL-6, with expression levels that substantially exceed those of MHC-II^+^ myeloid cells (55). Interestingly, IL-6 expression may be a feature of specific intestinal stromal cell subsets with distinct ontogeny or activation states. For example, human OSMR^high^ intestinal stromal cells were found to be enriched in IL-6 expression relative to their OSMR^low^ counterparts (55), consistent with the well-established ability of OSM to induce IL-6 expression in mesenchymal cells (78–86). Single-cell RNA-sequencing has similarly revealed substantial heterogeneity in the intestinal stromal cell compartment. High IL-6 expression is enriched in a stromal cell subset that is rare in healthy individuals, but dramatically expanded in patients with inflammatory bowel disease (IBD) (87). Notably, these cells were further characterized by expression of a variety of additional immunostimulatory molecules, including IL-33 and the FRC-associated chemokines CCL19 and CCL21, implying a specialized role in immune regulation (87). Notably, a disease-associated single nucleotide polymorphism (SNP) in the human *IL6* promoter was shown to control production of IL-6 by fibroblasts, but had no effect on IL-6 expression by CD14^+^ monocytes, suggesting that host genetics can also play an important role in determining stromal IL-6 output (88).

Following initiation of acute inflammation, IL-6 can act on several cell types to shape the quality of the ensuing immune response. For example, IL-6 controls the balance between inducible regulatory T cell (Treg) and Th17 differentiation following activation of naïve CD4^+^ T cells (41). Although stromal cells have not conclusively been demonstrated to contribute to this process, FRC-derived IL-6 has been suggested to support the development and maintenance of B cell responses. Medullary FRC were shown to be important regulators of plasma cell homeostasis, in part by producing the plasma cell survival factor IL-6 (89, 90). IL-6 is also necessary for the differentiation of follicular helper T cells (T_FH_), which drive the maturation of B cells and the generation of protective antibody responses (91, 92). Importantly, IL-6 induces production of IL-21 by T_FH_ cells, which is critical for both T_FH_ maintenance and plasma cell differentiation in germinal centers (93, 94). Publicly available data provided by the ImmGen project suggest that FRC constitutively express IL-6, and do so at levels that far exceed those of other lymph node-resident cell types (95). Thus, FRC-derived IL-6 is likely to be a central linchpin in the regulation of both T cell and B cell responses in secondary lymphoid organs.

In inflamed peripheral tissues, IL-6 controls the temporal switch from recruitment of granulocytes to preferential recruitment of mononuclear cells by modulating chemokine and cytokine production in local mesenchymal cells, including the suppression of TNF and IL-1β production, possibly via STAT3-mediated repression of NF-κB signaling (96, 97). IL-6 promotes the differentiation of monocytes into macrophages rather than dendritic cells *in vitro*, but genetic IL-6 deficiency does not affect dendritic cell frequencies *in vivo* (98–101). However, IL-6 appears to mediate alternative macrophage differentiation *in vivo* and inhibits inflammatory cytokine production and microbicidal activity by macrophages (102–105). IL-6 can also promote survival and regeneration of damaged epithelia during inflammatory challenges, a feature that can be subverted to promote cancer progression (106). Thus, while IL-6 is important for initiation of immune responses, it also promotes resolution of inflammation and tissue repair (54, 62). Notably, IL-6 protects mice from the lethal inflammatory effects of Staphylococcal enterotoxin B (SEB; a model of toxic shock), in direct contrast with TNF (107).

Although IL-6 is an important regulator of physiological immune responses, excess or chronic IL-6 production can promote inflammatory or fibrotic pathology. IL-6 has been implicated in a variety of inflammatory diseases, but is perhaps best studied in the context of arthritis, a condition that can be effectively treated via blockade of IL-6 signaling (42). Synovial fibroblasts in inflamed joints are thought to be the major source of IL-6, which is likely produced in response to a variety of inflammatory factors including TNF, IL-1β, LIF, and CDH11 (47, 53, 63). IL-6 is necessary for pathology in pre-clinical models of antigen-induced and spontaneous arthritis, in which it orchestrates a variety of inflammatory processes including activation of CCL2 production by synovial fibroblasts, differentiation of autoinflammatory Th17 cells, and bone erosion via increased osteoclastogenesis (108–111). IL-6 has also been shown to promote CCL20 production by fibroblasts, which may further promote recruitment of inflammatory Th17 cells (112). IL-6 appears to promote arthritis primarily via *trans* signaling, likely because synovial fibroblasts and activated CD4^+^ T cells do not express sufficient IL-6R to respond to IL-6 alone (108, 109). Indeed, CCL2 production following IL-6 stimulation of synovial fibroblasts requires the presence of soluble IL-6R, or a chimeric IL-6/IL-6R protein known as “hyper-IL-6” (108). IL-6 has also been shown to mediate fibrosis in the skin, lung, and heart (113–116). Notably, in a phase 2 clinical trial of patients with systemic sclerosis, a disease characterized by skin fibrosis, treatment with actemra (tocilizumab; anti-IL6R) dramatically attenuated fibrotic behavior and transcriptional signatures in dermal fibroblasts, along with significant attenuation of disease severity (113).

## Regulation of Inflammation and Hematopoiesis by the OSM-stromal Cell Axis

OSM is a pleiotropic cytokine with roles reported in a plethora of homeostatic and disease settings (34, 56, 65). Unlike IL-6, OSM is not generally produced at significant levels by stromal cells, but is instead a product of various hematopoietic cell types, including monocytes, macrophages, dendritic cells, neutrophils, eosinophils, mast cells, and T cells (34, 56, 65). OSM is further distinguished from IL-6 by the cellular distribution of its specific receptors (OSMR and LIFR in humans; OSMR in mice), which are largely restricted to non-hematopoietic cell types, notably epithelial cells, fibroblasts, endothelial cells, adipocytes, and neurons (34, 56, 65). OSM thus provides a means for leukocytes to deliver information to non-hematopoietic cells in inflamed or damaged tissues.

While little is known about the role of OSM in infectious disease or other host-defense settings, OSM can clearly influence hematopoietic homeostasis. OSM is necessary for the maintenance of granulocyte-macrophage, erythroid, megakaryocyte, and multipotential hematopoietic progenitor populations in the bone marrow, an effect that likely involves stimulation of bone marrow stromal cells by OSM (117–120). The ability of OSM to drive expression of CXCL12 (SDF1) in stromal cells may partly explain its effects on hematopoiesis (119, 121–123). However, aging studies have shown that OSM-deficient mice develop progressive hematological defects that include reduced numbers of circulating leukocytes, erythrocytes, and platelets, along with pronounced bone marrow adiposity. OSM was shown to suppress adipose differentiation of murine PDGFRα^+^ Sca1^+^ mesenchymal stem cells, thereby preventing the development of “fatty” marrow and safeguarding the hematopoietic niche (120). Several additional studies have confirmed that OSM acts on stromal progenitors to suppress adipocyte differentiation in favor of osteoblast development (124–130), suggesting that OSM plays a fundamental role in regulating the bone marrow microenvironment. Notably, overexpression of OSM in bone marrow stromal cells promotes the development of lethal myeloproliferative neoplasms and bone marrow fibrosis in mice (131, 132).

Numerous studies have implicated OSM in the pathogenesis of inflammatory conditions, including arthritis, inflammatory bowel disease, psoriasis, and allergic airway inflammation. Intra-articular adenoviral delivery of OSM causes arthritis-like pathology characterized by robust leukocyte infiltration, synovial hyperplasia, and erosion of bone and cartilage (133–135). Consistent with these findings, antibody blockade of OSM can reduce pathology in the collagen-induced and pristane-challenge pre-clinical models of inflammatory arthritis (136). Synovial fibroblasts respond to OSM by producing a wide variety of inflammatory factors including cytokines (e.g., IL-6), chemokines, (e.g., CCL2, CCL13, CXCL1), and leukocyte adhesion factors such as ICAM-1 (78, 82, 83, 134, 137–139). Furthermore, cytokine receptors such as IL1R1 (IL-1 receptor, type 1), gp130, and OSMR are induced by OSM, suggesting that OSM can sensitize synovial fibroblasts to additional cytokine stimulation. OSM can synergize with TNF and IL-1β to promote increased cytokine and chemokine expression, as well as high MMP (matrix metalloprotease) to TIMP1 (tissue inhibitor of metalloproteases) ratios to promote tissue damage. Remarkably, OSM alone drives high TIMP1 expression (134, 135, 138, 140, 141), and OSM only promotes net tissue degradation when acting in synergy with TNF, IL-1β, or IL-17A, suggesting that its pathogenicity may depend on the presence of other inflammatory factors (135, 137, 138, 142).

Emerging data suggest an important role for OSM-stromal cell interactions in barrier tissues, such as skin and intestinal mucosa. Dermal fibroblasts express extracellular matrix components such as collagens and glycosaminoglycans in response to OSM, and display an interferon-like response featuring upregulation of the viral RNA sensors RIG-I and MDA5 (143–146). While sufficient to induce skin inflammation, OSM may not be required for psoriasis-like pathology, as it is dispensable in the aldara (imiquimod) challenge model of psoriasis (55, 147, 148). OSM and OSMR are also overexpressed in the lesional skin of patients with atopic dermatitis, but whether OSM signaling is required for pathogenesis of this condition is unclear (147). A potentially non-redundant inflammatory role for OSM has been described for IBD, however. Unlike other barrier tissues such as the skin, normal epithelial cells do not express appreciable amounts of OSMR in either the mouse or human intestine (55). Intestinal fibroblasts, however, are highly sensitive to OSM and express a range of inflammatory factors in response to OSM stimulation, including IL-6, CCL2, CXCL10, and ICAM1. Like synovial fibroblasts, intestinal stromal cells show a synergistic inflammatory response to combined OSM and TNF treatment (55). Notably, genetic OSM deficiency or OSM blockade using an OSMR-gp130 fusion protein attenuates colitis in a dysbiosis-driven model of IBD that is refractory to anti-TNF therapy. Furthermore, OSM, OSMR, and stromal OSM response genes are highly predictive of resistance to anti-TNF therapy (e.g., infliximab and golimumab) in IBD patient cohorts, suggesting that high OSM expression drives an inflammatory axis that is mechanistically distinct from that of TNF (55). Intriguingly, OSM was not found to affect the early acute kinetics of colitis induction, but instead interfered with the resolution of inflammation, thereby contributing to disease chronicity and cumulative tissue damage. Experiments involving adenoviral overexpression of OSM in models of acute chemically induced colitis have yielded conflicting results, suggesting that the impact of OSM in these systems may be context dependent (149).

Consistent with data from studies of skin and joint inflammation, exogenous OSM promotes a robust inflammatory and tissue remodeling response in the lung (150–153), and blockade of OSM reduces disease severity in a mouse model of asthmatic airway inflammation (154). OSM stimulation of lung tissue induces a pronounced eosinophilia driven by OSM-induced expression of eotaxin (CCL11) in lung fibroblasts (152, 155). Unusually for an IL-6 family cytokine, OSM was shown to activate STAT6 in lung fibroblasts and activate CCL11 expression in synergy with IL-4 (155). OSM can also synergize with IL-4 to promote VCAM-1 expression by lung fibroblasts, which may promote increased eosinophil adhesion (156). Interestingly, the OSM-induced response in mouse lung tissue appears to differ between inbred strains. While intratracheal OSM promoted similar fibrotic changes in the lungs of BALB/c and C57BL/6 mice, only C57BL/6 animals induced an inflammatory Th2 response (151). Strain-dependent effects are known to occur in other inflammatory disease settings such models of colitis, in which C57BL/6 mice often display differing disease susceptibility compared to animals from the BALB/c, 129.SvEv, or C3H/HeJBir backgrounds (157–165). This demonstrates the importance of considering strain-dependent effects when interpreting *in vivo* studies of OSM or other IL-6 family cytokines. Furthermore, such findings emphasize the challenges inherent in predicting the biology of humans (an outbred population) based on mouse model data. It is noteworthy that single nucleotide polymorphisms in the *OSM* and *OSMR* genetic loci are associated with risk of developing IBD (*OSM* and *OSMR*) and the IgA nephropathy form of glomerulonephritis (*OSM*), but the functional significance of these risk variants is not yet understood (166–168).

## LIF and IL-11: Stromal Factors with Immunoregulatory and Fibrotic Properties

Unlike OSM, stromal cells appear to be important physiological sources of LIF and IL-11, both of which are produced in response to TGFβ stimulation (62, 66, 68, 169). While some studies have suggested pro-inflammatory roles for these cytokines (63, 170, 171), substantial evidence suggests that they also exert important anti-inflammatory effects. Treatment with recombinant LIF was demonstrated over 20 years ago to protect mice from mortality and to reduce systemic TNF production in models of endotoxin-induced septic shock (172). Similar findings were later reported in LIF-deficient (*Lif* ^−/−^) mice. Compared to wild type mice, *Lif* ^−/−^ animals had significantly greater mortality after endotoxin challenge, with dramatically increased levels of circulating TNF and IL-6 and reduced levels of IL-10, suggesting a potent anti-inflammatory effect (173). Treatment of mice and rats with recombinant IL-11 similarly reduced mortality, organ damage, and systemic cytokine production (TNF, IL-1β, and IFN-γ) in models of acute endotoxemia (174, 175). Recombinant IL-11 treatment likewise prevented T cell-driven liver injury in response to concanavalin-A, and protected mice from necrotizing pancreatitis following challenge with cerulein (176, 177).

Both LIF and IL-11 are reported to influence CD4^+^ T cell differentiation, favoring Treg development and Th2 development, respectively. Whereas IL-6 suppresses Treg development and promotes Th17 differentiation in synergy with TGFβ, LIF appears to have the opposite effect and can directly oppose the pro-Th17 activity of IL-6, possibly through induction of the negative regulatory factor SOCS3 (178, 179). Notably, LIF and IL-6 were identified as factors in human ovarian cancer ascites fluid that promoted differentiation of monocytes into anti-inflammatory macrophages with low IL-12 and high IL-10 production (100). *In vitro* culture models of human and mouse CD4^+^ T cells have demonstrated that IL-11 represses Th1 polarization and promotes expression of IL-4, IL-5, and IL-10, which are associated with Th2 immunity (180, 181). IL-11 was also shown to inhibit IL-12 production from monocytes, which likely further attenuates Th1 development (180). IL-11 treatment was highly effective for preventing mortality in a mouse model of graft-vs.-host disease, in which it blocked expression of Th1-related cytokines (IL-12, IL-2, and IFN-γ), and induced production of IL-4 (182). Consistent with this Th2-promoting effect, IL-11 signaling is necessary for airway inflammation in mice challenged with inhaled OVA antigen or transgenic animals that overexpress IL-13 in the lung (170, 183). Following OVA challenge, *Il11ra*^−/−^ mice showed dramatic reductions in lung-infiltrating Th2 cells, IL-13 expression, IgE production, and eosinophilia (170). Thus, while IL-11 can attenuate acute inflammation and Th1 responses, it may be pathogenic in conditions driven by Th2-polarized immunity.

Although LIF and IL-11 appear to have host-protective anti-inflammatory properties in the context of acute inflammatory challenges, they may be deleterious in setting of chronic inflammation and tissue remodeling. Like IL-6, IL-11 can drive a STAT3-mediated regenerative program in epithelial cells, a function that promotes malignancy in cancers of the intestine, stomach, and mammary gland (61, 184–187). A recent study made the surprising observation that synovial fibroblasts express LIF in response to inflammatory cytokines such as TNF and IL-17A, and autocrine LIF signaling drives STAT4, which synergizes with TNF/IL-17A-derived signals (NF-κB and C/EBPβ) to promote potent expression of IL-6 (63). LIF may therefore be an important driver of pathogenic IL-6 production in patients with rheumatoid arthritis. In cancer, LIF has been shown to activate cancer-associated fibroblasts to promote contractility, extracellular matrix remodeling, and cancer cell invasiveness (62, 66). Several studies have examined developmental abnormalities in transgenic mice that express either LIF or bovine OSM (a LIFR ligand in mice) downstream of the *Lck* promoter. These mice display a dramatic re-organization of lymph node structure, extrathymic T cell development, and myelosclerosis, suggesting that chronic overproduction of LIF in hematopoietic organs can have profound consequences on hematopoiesis (188–194). Transgenic overexpression of IL-11 in mouse lung tissue provokes lymphocyte and myeloid infiltration and considerable subepithelial fibrosis (171). IL-11 was also shown to be mitogenic for lung fibroblasts isolated from either healthy donors or those with idiopathic pulmonary fibrosis (IPF). More recently, two studies that incorporated both human cell culture systems and pre-clinical mouse models highlighted the importance of IL-11 for fibrosis in the lung, heart, and kidney (68, 169). Intriguingly, IL-11 production in primary fibroblasts was shown to be induced by various pro-fibrotic stimuli (including PDGF, TGFβ, IL-13, and OSM), and was critical for the adoption of a fibrogenic phenotype, suggesting that IL-11 mediates fibrosis in response to a wide variety of upstream stimuli (68, 169).

## Stromal Connections With IL-27

As noted above, IL-27 differs structurally from other IL-6 family members, being a heterodimer comprised of IL-27p28 and EBI3 (19). IL-27 can be thought of as an IL-6 family member primarily because it depends on gp130 as a receptor component (28, 37, 195). However, the structure of IL-27 is more comparable to that of IL-12 family members, all of which are similarly composed of heterodimers; IL-12 is comprised of IL-12p35 and IL-12p40, IL-23 of IL-12p40 and IL-23p19, and IL-35 of IL-12p35 and EBI3 (37, 195). Compared to its siblings in the IL-6 family, relatively little is known about the physiological interactions between IL-27 and stromal cells. In general, IL-27 has been studied in the context of communication between leukocyte populations, in which IL-27 can act in either an immunostimulatory or immunoregulatory capacity (37). For example, IL-27 synergizes with IL-12 to promote T-bet and IFN-γ expression by CD4^+^ T cells, thus promoting Th1 polarization (19, 196–199). However, IL-27 can also drive differentiation of T-bet expressing regulatory T cells (200), an effect that is in direct contrast to IL-6, which suppresses Treg differentiation. In both mice and humans, IL-27 is also thought to be a key driver of the immunoregulatory Tr1 subset of CD4^+^ T cells, which specializes in IL-10 production to suppress inflammation (201–204). Because the well-studied hematopoietic effects of IL-27 are beyond the scope of this discussion, the reader is referred to recent reviews of the subject (37, 195).

IL-27 can directly influence stromal cells, although studies of this process are scarce. Like OSM, IL-27 can induce cytokine and chemokine production by fibroblasts from the synovium and lung, and does so in synergy with TNF or IL-1β (70, 71). Factors induced in this manner include IL-6, CCL2, CXCL10, and ICAM-1 (70, 71). IL-27 has also been shown to enhance the sensitivity of pulmonary fibroblasts to lipopolysaccharide by promoting TLR4 expression (73). Finally, high circulating IL-27 concentrations are observed in patients with systemic sclerosis, and IL-27 stimulation of skin fibroblasts induced proliferation and collagen synthesis, implying a possible role for IL-27 in the fibrotic pathology of this disease (72). Thus, although IL-27 is best studied in the context of leukocyte-leukocyte interactions, it has the potential to regulate processes beyond those of the hematopoietic system through direct effects on stromal populations. A key challenge of future studies will be to determine whether IL-27 signaling in the stroma is functionally important *in vivo*.

## Effects of IL-6 Family Cytokines on Other Non-Hematopoietic Cell Types

This review has dealt specifically with the impact of IL-6 family cytokines on a narrow group of mesenchymal cells that collectively include various phenotypes of fibroblasts. However, it should be noted that several other non-hematopoietic cell types can respond strongly to IL-6 family members and likely mediate at least some of their effects *in vivo*. Such populations include endothelial cells, adipocytes, muscle cells, chondrocytes, epithelial cells, and glial cells. Importantly, the functional effects of IL-6 family cytokines on fibroblasts overlap broadly with those reported for other cell types, suggesting a degree of functional conservation between non-hematopoietic populations. For example, both OSM and IL-6 are implicated as potent drivers of endothelial activation; in response to these cytokines, endothelial cells upregulate expression of chemokines and cytokines that include CCL2 and IL-6, as well as adhesion factors such as ICAM-1, VCAM-1, P-selectin, and E-selectin (79, 205–208). In this fashion, OSM and IL-6 are thought to promote leukocyte recruitment to inflamed tissues via stimulation of the endothelium. Because OSM and IL-6 exert similar effects on stromal cells, it is possible that these cytokines control the duration and intensity of inflammatory responses through coordinated action on multiple non-hematopoietic cell types. OSM similarly induces chemokine and cytokine production in chondrocytes, osteoblasts, and smooth muscle cells (80, 85, 86, 209, 210).

The relative importance of these different cell populations for the execution of IL-6 family effects is poorly understood and probably context-dependent. For example, OSMR is highly expressed only by stromal cells in the intestinal mucosa, and it appears likely that these are the major mediators of OSM biology in this tissue (55). In contrast, OSMR is expressed broadly in many other tissues, making the identification of cell-type specific roles for OSM in these sites more challenging (34, 56, 65, 211). The ability of IL-6 and IL-11 to signal via both classical and *trans* mechanisms adds further complexity, since potential responder cells require only the ubiquitously expressed gp130 to recognize the *trans* forms of these cytokines. As such, we know little about the cell type requirements for IL-6/IL-11 functions *in vivo*, particularly under conditions of inflammatory pathology (18, 41). Finally, the ability of IL-6 family cytokines to modulate responses to other inflammatory mediators (e.g., TNF, IL-1β, IL-17A, IL-4) means that their effects *in vivo* likely depend on the composition of the broader cytokine milieu, as well as the relative abundance of cell types capable of receiving signals from both IL-6 family members and their synergy partners. Beyond characterizing the “receivers” of IL-6 family cytokines, we are similarly limited in our understanding of their *in vivo* sources. For example, IL-6 has traditionally been thought of as a primarily leukocyte-derived factor, but this concept is challenged by our growing awareness of non-hematopoietic cell types as sources of this cytokine (as well as factors such as LIF and IL-11). There is thus a clear need for carefully constructed *in vivo* studies that abrogate expression of individual IL-6 family receptors or ligands in specific cell types. This is increasingly feasible due to the growing availability of transgenic mice with floxed alleles of IL-6 family members and cell type-restricted Cre recombinase. Single-cell RNA-sequencing technology will also be a powerful tool to deconvolute the IL-6 family network *in vivo*.

## Targeting IL-6 Family Cytokines in the Clinic

IL-6 is well established as a valuable clinical target, and antibodies that block either IL-6 (e.g., siltuximab) or IL-6R (e.g., tocilizumab or sarilumab) are routinely used for treatment of inflammatory arthritis, juvenile idiopathic arthritis, multi-centric Castleman disease, cytokine release syndrome (commonly encountered in the setting of tumor immunotherapy), and giant cell arteritis (212–217). Despite these successes, clinical development of agents that target other members of the IL-6 family has been modest [reviewed here (18)]. A humanized anti-OSM monoclonal antibody (GSK315234) was tested in a phase II trial of rheumatoid arthritis and found to be well tolerated, but without significant clinical activity (218). However, the limited efficacy of this antibody was ascribed to its relatively weak target affinity when compared to the native OSM receptor complex, and clinical studies with a next-generation high-affinity anti-OSM antibody are currently underway, focused this time on systemic sclerosis (ClinicalTrials.gov Identifier: NCT03041 025). Based on its immunoregulatory properties, a trial of recombinant IL-11 in rheumatoid arthritis was also conducted, but without success (219).

A concept that emerges from the previous sections of this review is the substantial degree of functional similarity and cross-regulation between IL-6 family members. As such, targeting a single member of the IL-6 family for treatment of aetiologically complex diseases may be insufficient to fully engage the biology of interest. For example, although many patients with arthritis benefit from anti-IL6R therapy, this benefit is usually restricted to an incomplete decrease in disease activity, suggesting that other inflammatory pathways are in play (213, 216). Indeed, virtually all members of the IL-6 family (IL-6, OSM, LIF, IL-11, and IL-27) have been implicated as drivers of fibrosis. This raises the possibility that combinatorial blockade of two or more factors may be necessary to effectively neutralize IL-6 family biology in a given disease setting. Although clinical experience with JAK inhibitors (which block signaling from broad spectra of cytokines) demonstrates that simultaneous targeting of multiple cytokine pathways can be achieved (220), the efficacy of targeting multiple IL-6 family members would nevertheless require careful balancing against potential unexpected toxicities, particularly in the case of antibodies with slow rates of systemic clearance. The probability of success with such approaches will likely be increased by a combination of biomarker-guided patient stratification [e.g., OSM expression in IBD (55)] and the careful selection of combination candidates based on gene expression patterns in human tissues and mechanistic insights derived from carefully conducted pre-clinical studies.

## Concluding Remarks

**Figure 3:**
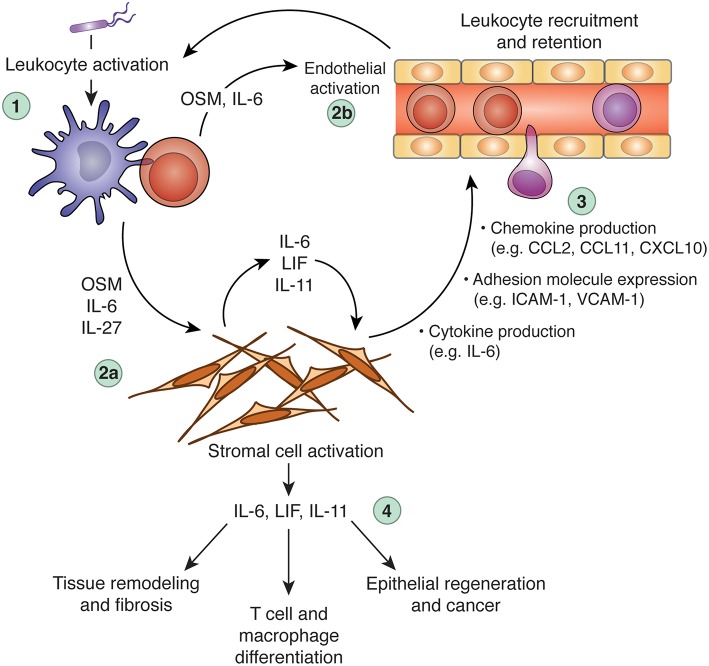
Regulation of inflammation and tissue repair processes via cross-talk between leukocytes, the IL-6 family, and stromal cells. Dysregulation of IL-6 family cytokine expression can promote chronic inflammation, tissue remodeling, and fibrosis. In a hypothetical scenario of acute inflammation, an initial inflammatory insult (e.g., microbial sensing) triggers leukocyte activation and production of inflammatory mediators, including OSM, IL-6, and IL-27 (step 1). These cytokines can act alone or in synergy with other inflammatory mediators (e.g., TNF, IL-1β, IL-17A, etc.) to activate tissue-resident stromal cells (step 2a) and the local vasculature (step 2b). Stromal cells stimulated by IL-6 family members produce a wide array of additional inflammatory mediators and adhesion molecules, including IL-6, CCL2, CXCL10, and ICAM-1. These stroma-derived factors collectively promote leukocyte recruitment and retention (step 3), to potentiate the inflammatory response. Stromal cells stimulated by leukocyte-derived IL-6, OSM, or IL-27 (particularly in combination with other inflammatory cytokines) can produce IL-6 family cytokines in turn, including IL-6, LIF, and IL-11, which can further stimulate stromal cells through autocrine feedback or act on additional cell types to modulate leukocyte behavior (e.g., T cell polarization), tissue remodeling (e.g., matrix deposition), and tissue regeneration (step 4). When appropriately regulated, these processes constitute beneficial responses to tissue injury or infectious challenge. When dysregulated, however, these processes can become self-sustaining, leading to chronic inflammation and associated pathology. CCL, C-C motif chemokine ligand; CXCL10, C-X-C motif chemokine ligand 10; IL, interleukin; ICAM-1, intercellular adhesion molecule 1; LIF, leukemia inhibitor factor; OSM, oncostatin M; VCAM-1, vascular cell adhesion molecule 1.

Increasing evidence indicates that stromal cells are integral to the biology of IL-6 family cytokines, both in healthy and pathological scenarios. Stromal cells both produce and sense IL-6 family cytokines, and can therefore not only respond to leukocyte-derived signals (e.g., OSM or IL-27), but also instruct leukocyte behavior (e.g., by producing IL-6, IL-11, or LIF) to influence the course of immunological processes (Figure 3). Although it is clear that IL-6 family cytokines play diverse roles *in vivo*, it is rare to identify specific cell types that are responsible for either cytokine production or cytokine responses. As such, although it is apparent that interactions between IL-6 family cytokines and stromal cells occur, whether these interactions are necessary *in vivo* is generally unclear. For example, while stromal cells appear to be important sources of IL-6 in models of inflammatory disease, whether they are indispensable sources of IL-6 has not been formally demonstrated. Similarly, while it is likely that stromal cells are key mediators of OSM biology in the intestine, the possibility that other mesenchymal cell types (or a hitherto unknown hematopoietic cell population) are involved cannot be ruled out. Greater use of genetic tools in animal studies, such as cell-type specific deletion of specific cytokine or cytokine receptor genes, is required to draw a more accurate roadmap of the functional connections between IL-6 family cytokines, individual cell types, and distinct biological processes. Rendering this map in greater detail will be critical for translating our knowledge of IL-6 family biology into clinical benefit.

## Author Contributions

NW conceived and wrote the manuscript, and prepared figures for this review.

### Conflict of Interest Statement

NW is an employee of Genentech, Inc, and is an inventor on patents related to oncostatin M.

## References

[1] Owens BMJ. Inflammation, innate immunity, and the intestinal stromal cell niche: opportunities and challenges. Front Immunol. (2015) 6:319. 10.3389/fimmu.2015.00319 26150817 PMC4471728

[2] Buechler MB Turley SJ. A short field guide to fibroblast function in immunity. Semin Immunol. (2018) 35:48–58. 10.1016/j.smim.2017.11.001 29198601

[3] Farr AG Berry ML Kim A Nelson AJ Welch MP Aruffo A. Characterization and cloning of a novel glycoprotein expressed by stromal cells in T-dependent areas of peripheral lymphoid tissues. J Exp Med. (1992) 176:1477–82. 10.1084/jem.176.5.1477 1402691 PMC2119410

[4] Fletcher AL Acton SE Knoblich K. Lymph node fibroblastic reticular cells in health and disease. Nature. (2015) 15:350–61. 10.1038/nri3846 25998961 PMC5152733

[5] Astarita JL Acton SE Turley SJ. Podoplanin: emerging functions in development, the immune system, and cancer. Front Immunol. (2012) 3:283. 10.3389/fimmu.2012.00283 22988448 PMC3439854

[6] Neyt K Perros F GeurtsvanKessel CH Hammad H Lambrecht BN. Tertiary lymphoid organs in infection and autoimmunity. Trends Immunol. (2012) 33:297–305. 10.1016/j.it.2012.04.006 22622061 PMC7106385

[7] Buckley CD Barone F Nayar S Bénézech C Caamaño J. Stromal cells in chronic inflammation and tertiary lymphoid organ formation. Annu Rev Immunol. (2015) 33:715–45. 10.1146/annurev-immunol-032713-120252 25861980

[8] Owens BMJ Simmons A. Intestinal stromal cells in mucosal immunity and homeostasis. Mucosal Immunol. (2013) 6:224–34. 10.1038/mi.2012.125 23235744

[9] Dakin SG Coles M Sherlock JP Powrie F Carr AJ Buckley CD. Pathogenic stromal cells as therapeutic targets in joint inflammation. Nat Rev Rheumatol. (2018) 14:714–26. 10.1038/s41584-018-0112-7 30420750

[10] Khan O Headley M Gerard A Wei W Liu L Krummel MF. Regulation of T cell priming by lymphoid stroma. PLoS ONE. (2011) 6:e26138. 10.1371/journal.pone.0026138 22110583 PMC3215700

[11] Siegert S Huang H-Y Yang C-Y Scarpellino L Carrie L Essex S. Fibroblastic reticular cells from lymph nodes attenuate T cell expansion by producing nitric oxide. PLoS ONE. (2011) 6:e27618. 10.1371/journal.pone.0027618 22110693 PMC3215737

[12] Lukacs-Kornek V Malhotra D Fletcher AL Acton SE Elpek KG Tayalia P. Regulated release of nitric oxide by nonhematopoietic stroma controls expansion of the activated T cell pool in lymph nodes. Nat Immunol. (2011) 12:1096–104. 10.1038/ni.2112 21926986 PMC3457791

[13] Tykocinski L-O Lauffer AM Bohnen A Kaul N-C Krienke S Tretter T. Synovial fibroblasts selectively suppress Th1 cell responses through IDO1-mediated tryptophan catabolism. J Immunol. (2017) 198:3109–17. 10.4049/jimmunol.1600600 28264972

[14] Haniffa MA Wang X-N Holtick U Rae M Isaacs JD Dickinson AM. Adult human fibroblasts are potent immunoregulatory cells and functionally equivalent to mesenchymal stem cells. J Immunol. (2007) 179:1595–604. 10.4049/jimmunol.179.3.1595 17641026

[15] Wynn TA. Integrating mechanisms of pulmonary fibrosis. J Exp Med. (2011) 208:1339–50. 10.1084/jem.20110551 21727191 PMC3136685

[16] Piersma B Bank RA Boersema M. Signaling in fibrosis: TGF-β, WNT, and YAP/TAZ converge. Front Med. (2015) 2:59. 10.3389/fmed.2015.00059 26389119 PMC4558529

[17] Garbers C Hermanns HM Schaper F Müller-Newen G Grötzinger J Rose-John S. Plasticity and cross-talk of interleukin 6-type cytokines. Cytokine Growth Factor Rev. (2012) 23:85–97. 10.1016/j.cytogfr.2012.04.001 22595692

[18] Jones SA Jenkins BJ. Recent insights into targeting the IL-6 cytokine family in inflammatory diseases and cancer. Nat Rev Immunol. (2018) 18:773–89. 10.1038/s41577-018-0066-7 30254251

[19] Pflanz S Timans JC Cheung J Rosales R Kanzler H Gilbert J. IL-27, a heterodimeric cytokine composed of EBI3 and p28 protein, induces proliferation of naive CD4+ T cells. Immunity. (2002) 16:779–90. 10.1016/S1074-7613(02)00324-2 12121660

[20] Boulanger MJ Chow D-C Brevnova EE Garcia KC. Hexameric structure and assembly of the interleukin-6/IL-6 alpha-receptor/gp130 complex. Science. (2003) 300:2101–4. 10.1126/science.1083901 12829785

[21] Barton VA Hall MA Hudson KR Heath JK. Interleukin-11 signals through the formation of a hexameric receptor complex. J Biol Chem. (2000) 275:36197–203. 10.1074/jbc.M004648200 10948192

[22] Matadeen R Hon W-C Heath JK Jones EY Fuller S. The dynamics of signal triggering in a gp130-receptor complex. Structure. (2007) 15:441–8. 10.1016/j.str.2007.02.006 17437716 PMC1885967

[23] Liu J Modrell B Aruffo A Scharnowske S Shoyab M. Interactions between oncostatin M and the IL-6 signal transducer, gp130. Cytokine. (1994) 6:272–8. 10.1016/1043-4666(94)90023-X 8054483

[24] Gearing DP Comeau MR Friend DJ Gimpel SD Thut CJ McGourty J. The IL-6 signal transducer, gp130: an oncostatin M receptor and affinity converter for the LIF receptor. Science. (1992) 255:1434–7. 10.1126/science.1542794 1542794

[25] Mosley B De Imus C Friend D Boiani N Thoma B Park LS. Dual oncostatin M (OSM) receptors. Cloning and characterization of an alternative signaling subunit conferring OSM-specific receptor activation. J Biol Chem. (1996) 271:32635–43. 10.1074/jbc.271.51.32635 8999038

[26] Skiniotis G Lupardus PJ Martick M Walz T Garcia KC. Structural organization of a full-length gp130/LIF-R cytokine receptor transmembrane complex. Mol Cell. (2008) 31:737–48. 10.1016/j.molcel.2008.08.011 18775332 PMC2607196

[27] Gearing DP Thut CJ VandeBos T Gimpel SD Delaney PB King J. Leukemia inhibitory factor receptor is structurally related to the IL-6 signal transducer, gp130. EMBO J. (1991) 10:2839–48. 10.1002/j.1460-2075.1991.tb07833.x 1915266 PMC452994

[28] Pflanz S Hibbert L Mattson J Rosales R Vaisberg E Bazan JF. WSX-1 and glycoprotein 130 constitute a signal-transducing receptor for IL-27. J Immunol. (2004) 172:2225–31. 10.4049/jimmunol.172.4.2225 14764690

[29] Davis S Aldrich TH Stahl N Pan L Taga T Kishimoto T. LIFR beta and gp130 as heterodimerizing signal transducers of the tripartite CNTF receptor. Science. (1993) 260:1805–8. 10.1126/science.8390097 8390097

[30] Davis S Aldrich TH Valenzuela DM Wong VV Furth ME Squinto SP. The receptor for ciliary neurotrophic factor. Science. (1991) 253:59–63. 10.1126/science.1648265 1648265

[31] Sims NA. Cardiotrophin-like cytokine factor 1 (CLCF1) and neuropoietin (NP) signalling and their roles in development, adulthood, cancer and degenerative disorders. Cytokine Growth Factor Rev. (2015) 26:517–22. 10.1016/j.cytogfr.2015.07.014 26198769

[32] Lindberg RA Juan TS Welcher AA Sun Y Cupples R Guthrie B. Cloning and characterization of a specific receptor for mouse oncostatin M. Mol Cell Biol. (1998) 18:3357–67. 10.1128/MCB.18.6.3357 9584176 PMC108917

[33] Drechsler J Grötzinger J Hermanns HM. Characterization of the rat oncostatin M receptor complex which resembles the human, but differs from the murine cytokine receptor. PLoS ONE. (2012) 7:e43155. 10.1371/journal.pone.0043155 22937020 PMC3425591

[34] Hermanns HM. Oncostatin M and interleukin-31: Cytokines, receptors, signal transduction and physiology. Cytokine Growth Factor Rev. (2015) 26:545–58. 10.1016/j.cytogfr.2015.07.006 26198770

[35] Heinrich PC Behrmann I Haan S Hermanns HM Müller-Newen G Schaper F. Principles of interleukin (IL)-6-type cytokine signalling and its regulation. Biochem J. (2003) 374:1–20. 10.1042/bj20030407 12773095 PMC1223585

[36] Hermanns HM Radtke S Schaper F Heinrich PC Behrmann I. Non-redundant signal transduction of interleukin-6-type cytokines. J Biol Chem. (2000) 275:40742–8. 10.1074/jbc.M005408200 11016927

[37] Jones GW Hill DG Cardus A Jones SA. IL-27: a double agent in the IL-6 family. Clin Exp Immunol. (2018) 193:37–46. 10.1111/cei.13116 29437229 PMC6037998

[38] Rose-John S Heinrich PC. Soluble receptors for cytokines and growth factors: generation and biological function. Biochem J. (1994) 300(Pt 2):281–90. 10.1042/bj3000281 8002928 PMC1138158

[39] Peters M Jacobs S Ehlers M Vollmer P Müllberg J Wolf E. The function of the soluble interleukin 6 (IL-6) receptor *in vivo*: sensitization of human soluble IL-6 receptor transgenic mice towards IL-6 and prolongation of the plasma half-life of IL-6. J Exp Med. (1996) 183:1399–406. 10.1084/jem.183.4.1399 8666898 PMC2192475

[40] Jones SA Horiuchi S Topley N Yamamoto N Fuller GM. The soluble interleukin 6 receptor: mechanisms of production and implications in disease. FASEB J. (2001) 15:43–58. 10.1096/fj.99-1003rev 11149892

[41] Hunter CA Jones SA. IL-6 as a keystone cytokine in health and disease. Nat Immunol. (2015) 16:448–57. 10.1038/ni.3153 25898198

[42] Garbers C Heink S Korn T Rose-John S. Interleukin-6: designing specific therapeutics for a complex cytokine. Nat Rev Drug Discov. (2018) 17:395–412. 10.1038/nrd.2018.45 29725131

[43] Georganas C Liu H Perlman H Hoffmann A Thimmapaya B Pope RM Regulation of IL-6 and IL-8 expression in rheumatoid arthritis synovial fibroblasts: the dominant role for NF-kappa B but not C/EBP beta or c-Jun. J Immunol. (2000) 165:7199–206. 10.4049/jimmunol.165.12.7199 11120852

[44] Hosokawa I Hosokawa Y Ozaki K Yumoto H Nakae H Matsuo T. Proinflammatory effects of muramyldipeptide on human gingival fibroblasts. J Periodont Res. (2010) 45:193–9. 10.1111/j.1600-0765.2009.01217.x 20470259

[45] Kyburz D Rethage J Seibl R Lauener R Gay RE Carson DA Bacterial peptidoglycans but not CpG oligodeoxynucleotides activate synovial fibroblasts by toll-like receptor signaling. Arthritis Rheum. (2003) 48:642–50. 10.1002/art.10848 12632416

[46] Ospelt C Brentano F Jüngel A Rengel Y Kolling C Michel BA. Expression, regulation, and signaling of the pattern-recognition receptor nucleotide-binding oligomerization domain 2 in rheumatoid arthritis synovial fibroblasts. Arthritis Rheum. (2009) 60:355–63. 10.1002/art.24226 19180502

[47] Fossiez F Djossou O Chomarat P Flores-Romo L Ait-Yahia S Maat C. T cell interleukin-17 induces stromal cells to produce proinflammatory and hematopoietic cytokines. J Exp Med. (1996) 183:2593–603. 10.1084/jem.183.6.2593 8676080 PMC2192621

[48] Hata K Andoh A Shimada M Fujino S Bamba S Araki Y. IL-17 stimulates inflammatory responses via NF-kappaB and MAP kinase pathways in human colonic myofibroblasts. Am J Physiol Gastrointest Liver Physiol. (2002) 282:G1035–44. 10.1152/ajpgi.00494.2001 12016129

[49] Andoh A Hata K Araki Y Fujiyama Y Bamba T. Interleukin (IL)-4 and IL-17 synergistically stimulate IL-6 secretion in human colonic myofibroblasts. Int J Mol Med. (2002) 10:631–4. 10.3892/ijmm.10.5.631 12373306

[50] Shimada M Andoh A Hata K Tasaki K Araki Y Fujiyama Y. IL-6 secretion by human pancreatic periacinar myofibroblasts in response to inflammatory mediators. J Immunol. (2002) 168:861–8. 10.4049/jimmunol.168.2.861 11777983

[51] Katz Y Nadiv O Beer Y. Interleukin-17 enhances tumor necrosis factor α-induced synthesis of interleukins 1, 6, and 8 in skin and synovial fibroblasts: a possible role as a “fine-tuning cytokine” in inflammation processes. Arthritis Rheum. (2001) 44:2176–84. 10.1002/1529-0131(200109)44:9<2176::AID-ART371>3.0.CO;2-4 11592383

[52] Parsonage G Falciani F Burman A Filer A Ross E Bofill M. Global gene expression profiles in fibroblasts from synovial, skin and lymphoid tissue reveals distinct cytokine and chemokine expression patterns. Thrombosis Haemostasis. (2003) 90:688–97. 10.1160/TH03-04-0208 14515190

[53] Chang SK Noss EH Chen M Gu Z Townsend K Grenha R. Cadherin-11 regulates fibroblast inflammation. Proc Natl Acad Sci USA. (2011) 108:8402–7. 10.1073/pnas.1019437108 21536877 PMC3100978

[54] Dufour AM Alvarez M Russo B Chizzolini C. Interleukin-6 and type-I collagen production by systemic sclerosis fibroblasts are differentially regulated by interleukin-17A in the presence of transforming growth factor-beta 1. Front Immunol. (2018) 9:1865. 10.3389/fimmu.2018.01865 30150989 PMC6099180

[55] West NR Hegazy AN Owens BMJ Bullers SJ Linggi B Buonocore S Oncostatin M drives intestinal inflammation and predicts response to tumor necrosis factor-neutralizing therapy in patients with inflammatory bowel disease. Nature. (2017) 23:579–89. 10.1038/nm.4307 PMC5420447 28368383

[56] West NR Owens BMJ Hegazy AN. The oncostatin M-stromal cell axis in health and disease. Scand J Immunol. (2018) 88:e12694–18. 10.1111/sji.12694 29926972

[57] Eickelberg O Pansky A Mussmann R Bihl M Tamm M Hildebrand P. Transforming growth factor-beta1 induces interleukin-6 expression via activating protein-1 consisting of JunD homodimers in primary human lung fibroblasts. J Biol Chem. (1999) 274:12933–8. 10.1074/jbc.274.18.12933 10212284

[58] Coppé J-P Desprez P-Y Krtolica A Campisi J. The senescence-associated secretory phenotype: the dark side of tumor suppression. Annu Rev Pathol. (2010) 5:99–118. 10.1146/annurev-pathol-121808-102144 20078217 PMC4166495

[59] Valencia T Kim JY Abu-Baker S Moscat-Pardos J Ahn CS Reina-Campos M. Metabolic reprogramming of stromal fibroblasts through p62-mTORC1 signaling promotes inflammation and tumorigenesis. Cancer Cell. (2014) 26:121–35. 10.1016/j.ccr.2014.05.004 25002027 PMC4101061

[60] Lorgeot V Rougier F Fixe P Cornu E Praloran V Denizot Y. Spontaneous and inducible production of leukaemia inhibitory factor by human bone marrow stromal cells. Cytokine. (1997) 9:754–8. 10.1006/cyto.1997.0225 9344507

[61] Calon A Espinet E Palomo-Ponce S Tauriello DVF Iglesias M Céspedes MV. Dependency of colorectal cancer on a TGF-β-driven program in stromal cells for metastasis initiation. Cancer Cell. (2012) 22:571–84. 10.1016/j.ccr.2012.08.013 23153532 PMC3512565

[62] Albrengues J Bourget I Pons C Butet V Hofman P Tartare-Deckert S. LIF mediates proinvasive activation of stromal fibroblasts in cancer. Cell Rep. (2014) 7:1664–78. 10.1016/j.celrep.2014.04.036 24857661

[63] Nguyen HN Noss EH Mizoguchi F Huppertz C Wei KS Watts GFM. Autocrine loop involving IL-6 family member LIF, LIF receptor, and STAT4 drives sustained fibroblast production of inflammatory mediators. Immunity. (2017) 46:220–32. 10.1016/j.immuni.2017.01.004 28228280 PMC5567864

[64] Souza PPC Palmqvist P Lundberg P Lundgren I Hänström L Souza JAC. Interleukin-4 and interleukin-13 inhibit the expression of leukemia inhibitory factor and interleukin-11 in fibroblasts. Mol Immunol. (2012) 49:601–10. 10.1016/j.molimm.2011.10.009 22142941

[65] Richards CD. The enigmatic cytokine oncostatin M and roles in disease. ISRN Inflammation. (2013) 2013:1–23. 10.1155/2013/512103 24381786 PMC3870656

[66] Albrengues J Bertero T Grasset E Bonan S Maiel M Bourget I. Epigenetic switch drives the conversion of fibroblasts into proinvasive cancer-associated fibroblasts. Nat Commun. (2015) 6:10204. 10.1038/ncomms10204 26667266 PMC4682161

[67] Elshabrawy HA Volin MV Essani AB Chen Z McInnes IB Van Raemdonck K. IL-11 facilitates a novel connection between RA joint fibroblasts and endothelial cells. Angiogenesis. (2018) 21:215–28. 10.1007/s10456-017-9589-y 29327326 PMC5878720

[68] Schafer S Viswanathan S Widjaja AA Lim W-W Moreno-Moral A DeLaughter DM. IL-11 is a crucial determinant of cardiovascular fibrosis. Nature. (2017) 552:110–5. 10.1038/nature24676 29160304 PMC5807082

[69] Moodley YP Scaffidi AK Misso NL Keerthisingam C McAnulty RJ Laurent GJ. Fibroblasts isolated from normal lungs and those with idiopathic pulmonary fibrosis differ in interleukin-6/gp130-mediated cell signaling and proliferation. Am J Pathol. (2003) 163:345–54. 10.1016/S0002-9440(10)63658-9 12819039 PMC1868172

[70] Wong CK Chen DP Tam LS Li EK Yin YB Lam CWK. Effects of inflammatory cytokine IL-27 on the activation of fibroblast-like synoviocytes in rheumatoid arthritis. Arthritis Res Ther. (2010) 12:R129. 10.1186/ar3067 20604932 PMC2945019

[71] Dong S Zhang X He Y Xu F Li D Xu W. Synergy of IL-27 and TNF-α in regulating CXCL10 expression in lung fibroblasts. Am J Respir Cell Mol Biol. (2013) 48:518–30. 10.1165/rcmb.2012-0340OC 23333920

[72] Yoshizaki A Yanaba K Iwata Y Komura K Ogawa A Muroi E. Elevated serum interleukin-27 levels in patients with systemic sclerosis: association with T cell, B cell and fibroblast activation. Ann Rheum Dis. (2011) 70:194–200. 10.1136/ard.2009.121053 20705635

[73] Su Y Yao H Wang H Xu F Li D Li D. IL-27 enhances innate immunity of human pulmonary fibroblasts and epithelial cells through upregulation of TLR4 expression. Am J Physiol Lung Cell Mol Physiol. (2016) 310:L133–41. 10.1152/ajplung.00307.2015 26608531

[74] Wong C-K Leung KM-L Qiu H-N Chow JY-S Choi AOK Lam CW-K. Activation of eosinophils interacting with dermal fibroblasts by pruritogenic cytokine IL-31 and alarmin IL-33: implications in atopic dermatitis. PLoS ONE. (2012) 7:e29815. 10.1371/journal.pone.0029815 22272250 PMC3260155

[75] Jawa RS Chattopadhyay S Tracy E Wang Y Huntoon K Dayton MT. Regulated expression of the IL-31 receptor in bronchial and alveolar epithelial cells, pulmonary fibroblasts, and pulmonary macrophages. J Interferon Cytokine Res. (2008) 28:207–19. 10.1089/jir.2007.0057 18439099

[76] Chou DB Sworder B Bouladoux N Roy CN Uchida AM Grigg M. Stromal-derived IL-6 alters the balance of myeloerythroid progenitors during Toxoplasma gondii infection. J Leukocyte Biol. (2012) 92:123–31. 10.1189/jlb.1011527 22493080 PMC3382309

[77] Lagneaux L Delforge A Snoeck R Bosmans E Moreau JF Taupin JL. Human cytomegalovirus increases constitutive production of interleukin-6 and leukemia inhibitory factor by bone marrow stromal cells. Blood. (1996) 87:59–66. 8547677

[78] Richards CD Langdon C Botelho F Brown TJ Agro A. Oncostatin M inhibits IL-1-induced expression of IL-8 and granulocyte-macrophage colony-stimulating factor by synovial and lung fibroblasts. J Immunol. (1996) 156:343–9. 8598483

[79] Modur V Feldhaus MJ Weyrich AS Jicha DL Prescott SM Zimmerman GA. Oncostatin M is a proinflammatory mediator. *In vivo* effects correlate with endothelial cell expression of inflammatory cytokines and adhesion molecules. J Clin Invest. (1997) 100:158–68. 10.1172/JCI119508 9202068 PMC508176

[80] Schnittker D Kwofie K Ashkar A Trigatti B Richards CD. Oncostatin M and TLR-4 ligand synergize to induce MCP-1, IL-6, and VEGF in human aortic adventitial fibroblasts and smooth muscle cells. Mediat Inflamm. (2013) 2013:1–14. 10.1155/2013/317503 24307759 PMC3836373

[81] Ruprecht K Kuhlmann T Seif F Hummel V Kruse N Brück W. Effects of oncostatin M on human cerebral endothelial cells and expression in inflammatory brain lesions. J Neuropathol Exp Neurol. (2001) 60:1087–98. 10.1093/jnen/60.11.1087 11706938

[82] Le Goff B Singbrant S Tonkin BA Martin TJ Romas E Sims NA Oncostatin M acting via OSMR, augments the actions of IL-1 and TNF in synovial fibroblasts. Cytokine. (2014) 68:101–9. 10.1016/j.cyto.2014.04.001 24767864

[83] Migita K Komori A Torigoshi T Maeda Y Izumi Y Jiuchi Y. CP690,550 inhibits oncostatin M-induced JAK/STAT signaling pathway in rheumatoid synoviocytes. Arthritis Res Ther. (2011) 13:R72. 10.1186/ar3333 21548952 PMC3218881

[84] Hurst SM McLoughlin RM Monslow J Owens S Morgan L Fuller GM. Secretion of oncostatin M by infiltrating neutrophils: regulation of IL-6 and chemokine expression in human mesothelial cells. J Immunol. (2002) 169:5244–51. 10.4049/jimmunol.169.9.5244 12391243

[85] Faffe DS Flynt L Mellema M Whitehead TR Bourgeois K Panettieri RA. Oncostatin M causes VEGF release from human airway smooth muscle: synergy with IL-1beta. AJP Lung Cell Mol Physiol. (2005) 288:L1040–8. 10.1152/ajplung.00333.2004 15665043

[86] Kwofie K Scott M Rodrigues R Guerette J Radford K Nair P. Regulation of IL-17A responses in human airway smooth muscle cells by Oncostatin M. Respir Res. (2015) 16:14. 10.1186/s12931-014-0164-4 25849622 PMC4332894

[87] Kinchen J Chen HH Parikh K Antanaviciute A Jagielowicz M Fawkner-Corbett D. Structural remodeling of the human colonic mesenchyme in inflammatory bowel disease. Cell. (2018) 175:372–386.e17. 10.1016/j.cell.2018.08.067 30270042 PMC6176871

[88] Noss EH Nguyen HN Chang SK Watts GFM Brenner MB Genetic polymorphism directs IL-6 expression in fibroblasts but not selected other cell types. Proc Natl Acad Sci USA. (2015) 112:14948–53. 10.1073/pnas.1520861112 26578807 PMC4672776

[89] Huang H-Y Rivas-Caicedo A Renevey F Cannelle H Peranzoni E Scarpellino L. Identification of a new subset of lymph node stromal cells involved in regulating plasma cell homeostasis. Proc Natl Acad Sci USA. (2018) 115:E6826–35. 10.1073/pnas.1712628115 29967180 PMC6055158

[90] Zhang Y Tech L George LA Acs A Durrett RE Hess H. Plasma cell output from germinal centers is regulated by signals from Tfh and stromal cells. J Exp Med. (2018) 215:1227–43. 10.1084/jem.20160832 29549115 PMC5881458

[91] Vinuesa CG Linterman MA Yu D MacLennan ICM Follicular helper T cells. Annu Rev Immunol. (2016) 34:335–68. 10.1146/annurev-immunol-041015-055605 26907215

[92] Dienz O Eaton SM Bond JP Neveu W Moquin D Noubade R. The induction of antibody production by IL-6 is indirectly mediated by IL-21 produced by CD4+ T cells. J Exp Med. (2009) 206:69–78. 10.1084/jem.20081571 19139170 PMC2626667

[93] Ise W Kurosaki T. Plasma cell differentiation during the germinal center reaction. Immunol Rev. (2019) 288:64–74. 10.1111/imr.12751 30874351

[94] Wu H Deng Y Zhao M Zhang J Zheng M Chen G. Molecular control of follicular helper T cell development and differentiation. Front Immunol. (2018) 9:2470. 10.3389/fimmu.2018.02470 30410493 PMC6209674

[95] Heng TSP Painter MW Immunological Genome Project Consortium. The immunological genome project: networks of gene expression in immune cells. Nat Immunol. (2008) 9:1091–4. 10.1038/ni1008-1091 18800157

[96] Hurst SM Wilkinson TS McLoughlin RM Jones S Horiuchi S Yamamoto N. Il-6 and its soluble receptor orchestrate a temporal switch in the pattern of leukocyte recruitment seen during acute inflammation. Immunity. (2001) 14:705–14. 10.1016/S1074-7613(01)00151-0 11420041

[97] Zhang H Hu H Greeley N Jin J Matthews AJ Ohashi E. STAT3 restrains RANK- and TLR4-mediated signalling by suppressing expression of the E2 ubiquitin-conjugating enzyme Ubc13. Nat Commun. (2014) 5:5798. 10.1038/ncomms6798 25503582 PMC4270087

[98] Chomarat P Banchereau J Davoust J Palucka AK. IL-6 switches the differentiation of monocytes from dendritic cells to macrophages. Nat Immunol. (2000) 1:510–4. 10.1038/82763 11101873

[99] Chomarat P Dantin C Bennett L Banchereau J Palucka AK. TNF skews monocyte differentiation from macrophages to dendritic cells. J Immunol. (2003) 171:2262–9. 10.4049/jimmunol.171.5.2262 12928370

[100] Duluc D Delneste Y Tan F Moles M-P Grimaud L Lenoir J. Tumor-associated leukemia inhibitory factor and IL-6 skew monocyte differentiation into tumor-associated macrophage-like cells. Blood. (2007) 110:4319–30. 10.1182/blood-2007-02-072587 17848619

[101] Bleier JI Pillarisetty VG Shah AB DeMatteo RP. Increased and long-term generation of dendritic cells with reduced function from IL-6-deficient bone marrow. J Immunol. (2004) 172:7408–16. 10.4049/jimmunol.172.12.7408 15187118

[102] Mauer J Chaurasia B Goldau J Vogt MC Ruud J Nguyen KD. Signaling by IL-6 promotes alternative activation of macrophages to limit endotoxemia and obesity-associated resistance to insulin. Nat Immunol. (2014) 15:423–30. 10.1038/ni.2865 24681566 PMC4161471

[103] Silver JS Stumhofer JS Passos S Ernst M Hunter CA. IL-6 mediates the susceptibility of glycoprotein 130 hypermorphs to *Toxoplasma gondii*. J Immunol. (2011) 187:350–60. 10.4049/jimmunol.1004144 21606248 PMC3119722

[104] Beaman MH Hunter CA Remington JS. Enhancement of intracellular replication of *Toxoplasma gondii* by IL-6. Interactions with IFN-gamma and TNF-alpha. J Immunol. (1994) 153:4583–7. 7963530

[105] Nagabhushanam V Solache A Ting L-M Escaron CJ Zhang JY Ernst JD. Innate inhibition of adaptive immunity: *Mycobacterium tuberculosis*-induced IL-6 inhibits macrophage responses to IFN-gamma. J Immunol. (2003) 171:4750–7. 10.4049/jimmunol.171.9.4750 14568951

[106] Grivennikov S Karin E Terzic J Mucida D Yu G-Y Vallabhapurapu S. IL-6 and Stat3 are required for survival of intestinal epithelial cells and development of colitis-associated cancer. Cancer Cell. (2009) 15:103–13. 10.1016/j.ccr.2009.01.001 19185845 PMC2667107

[107] Barton BE Shortall J Jackson JV. Interleukins 6 and 11 protect mice from mortality in a staphylococcal enterotoxin-induced toxic shock model. Infect Immun. (1996) 64:714–8. 8641771 10.1128/iai.64.3.714-718.1996 PMC173827

[108] Nowell MA Richards PJ Horiuchi S Yamamoto N Rose-John S Topley N. Soluble IL-6 receptor governs IL-6 activity in experimental arthritis: blockade of arthritis severity by soluble glycoprotein 130. J Immunol. (2003) 171:3202–9. 10.4049/jimmunol.171.6.3202 12960349

[109] Nowell MA Williams AS Carty SA Scheller J Hayes AJ Jones GW. Therapeutic targeting of IL-6 trans signaling counteracts STAT3 control of experimental inflammatory arthritis. J Immunol. (2008) 182:613–22. 10.4049/jimmunol.182.1.613 19109195

[110] Hata H Sakaguchi N Yoshitomi H Iwakura Y Sekikawa K Azuma Y. Distinct contribution of IL-6, TNF-alpha, IL-1, and IL-10 to T cell-mediated spontaneous autoimmune arthritis in mice. J Clin Invest. (2004) 114:582–8. 10.1172/JCI200421795 15314695 PMC503774

[111] Wong PKK Quinn JMW Sims NA van Nieuwenhuijze A Campbell IK Wicks IP. Interleukin-6 modulates production of T lymphocyte-derived cytokines in antigen-induced arthritis and drives inflammation-induced osteoclastogenesis. Arthritis Rheum. (2006) 54:158–68. 10.1002/art.21537 16385511

[112] Walch-Rückheim B Mavrova R Henning M Vicinus B Kim Y-J Bohle RM. Stromal fibroblasts induce CCL20 through IL6/C/EBPβ to support the recruitment of Th17 cells during cervical cancer progression. Cancer Res. (2015) 75:5248–59. 10.1158/0008-5472.CAN-15-0732 26631268

[113] Denton CP Ong VH Xu S Chen-Harris H Modrusan Z Lafyatis R. Therapeutic interleukin-6 blockade reverses transforming growth factor-beta pathway activation in dermal fibroblasts: insights from the faSScinate clinical trial in systemic sclerosis. Ann Rheum Dis. (2018) 77:1362–71. 10.1136/annrheumdis-2018-213031 29853453 PMC6104680

[114] Le T-TT Karmouty-Quintana H Melicoff E Le T-TT Weng T Chen N-Y. Blockade of IL-6 trans signaling attenuates pulmonary fibrosis. J Immunol. (2014) 193:3755–68. 10.4049/jimmunol.1302470 25172494 PMC4169999

[115] Ma F Li Y Jia L Han Y Cheng J Li H. Macrophage-stimulated cardiac fibroblast production of IL-6 is essential for TGF β/Smad activation and cardiac fibrosis induced by angiotensin II. PLoS ONE. (2012) 7:e35144. 10.1371/journal.pone.0035144 22574112 PMC3344835

[116] Meléndez GC McLarty JL Levick SP Du Y Janicki JS Brower GL. Interleukin 6 mediates myocardial fibrosis, concentric hypertrophy, and diastolic dysfunction in rats. Hypertension. (2010) 56:225–31. 10.1161/HYPERTENSIONAHA.109.148635 20606113 PMC2921860

[117] Broxmeyer HE Bruns HA Zhang S Cooper S Hangoc G McKenzie ANJ. Th1 cells regulate hematopoietic progenitor cell homeostasis by production of oncostatin M. Immunity. (2002) 16:815–25. 10.1016/S1074-7613(02)00319-9 12121663

[118] Tanaka M Hirabayashi Y Sekiguchi T Inoue T Katsuki M Miyajima A. Targeted disruption of oncostatin M receptor results in altered hematopoiesis. Blood. (2003) 102:3154–62. 10.1182/blood-2003-02-0367 12855584

[119] Minehata K-I Takeuchi M Hirabayashi Y Inoue T Donovan P Tanaka M. Oncostatin M maintains the hematopoietic microenvironment and retains hematopoietic progenitors in the bone marrow. Int J Hematol. (2006) 84:319–27. 10.1532/IJH97.06090 17118758

[120] Sato F Miyaoka Y Miyajima A Tanaka M. Oncostatin M maintains the hematopoietic microenvironment in the bone marrow by modulating adipogenesis and osteogenesis. PLoS ONE. (2014) 9:e116209. 10.1371/journal.pone.0116209 25551451 PMC4281151

[121] Hoermann G Cerny-Reiterer S Sadovnik I Müllauer L Bilban M Gröger M. Oncostatin M is a FIP1L1/PDGFRA-dependent mediator of cytokine production in chronic eosinophilic leukemia. Allergy. (2013) 68:713–23. 10.1111/all.12139 23621172

[122] Hohensinner PJ Kaun C Rychli K Niessner A Pfaffenberger S Rega G. The inflammatory mediator oncostatin M induces stromal derived factor-1 in human adult cardiac cells. FASEB J. (2009) 23:774–82. 10.1096/fj.08-108035 19019853

[123] Lee MJ Song HY Kim MR Sung S-M Jung JS Kim JH. Oncostatin M stimulates expression of stromal-derived factor-1 in human mesenchymal stem cells. Int J Biochem Cell Biol. (2007) 39:650–9. 10.1016/j.biocel.2006.11.003 17169599

[124] Walker EC Johnson RW Hu Y Brennan HJ Poulton IJ Zhang J-G Murine oncostatin M acts via leukemia inhibitory factor receptor to phosphorylate signal transducer and activator of transcription 3 (STAT3) but not STAT1, an effect that protects bone mass. J Biol Chem. (2016) 291:21703–16. 10.1074/jbc.M116.748483 27539849 PMC5076839

[125] Walker EC McGregor NE Poulton IJ Solano M Pompolo S Fernandes TJ. Oncostatin M promotes bone formation independently of resorption when signaling through leukemia inhibitory factor receptor in mice. J Clin Invest. (2010) 120:582–92. 10.1172/JCI40568 20051625 PMC2810087

[126] Guihard P Danger Y Brounais B David E Brion R Delecrin J. Induction of osteogenesis in mesenchymal stem cells by activated monocytes/macrophages depends on oncostatin M signaling. Stem Cells. (2012) 30:762–72. 10.1002/stem.1040 22267310

[127] Guihard P Boutet M-A Royer BB-L Gamblin A-L Amiaud J Renaud A. Oncostatin M, an inflammatory cytokine produced by macrophages, supports intramembranous bone healing in a mouse model of tibia injury. Am J Pathol. (2015) 185:765–75. 10.1016/j.ajpath.2014.11.008 25559270

[128] Johnson RW Brennan HJ Vrahnas C Poulton IJ McGregor NE Standal T. The primary function of gp130 signaling in osteoblasts is to maintain bone formation and strength, rather than promote osteoclast formation. J Bone Miner Res. (2014) 29:1492–505. 10.1002/jbmr.2159 24339143

[129] Nicolaidou V Wong MM Redpath AN Ersek A Baban DF Williams LM. Monocytes induce STAT3 activation in human mesenchymal stem cells to promote osteoblast formation. PLoS ONE. (2012) 7:e39871–16. 10.1371/journal.pone.0039871 22802946 PMC3389003

[130] Fernandes TJ Hodge JM Singh PP Eeles DG Collier FM Holten I. Cord blood-derived macrophage-lineage cells rapidly stimulate osteoblastic maturation in mesenchymal stem cells in a glycoprotein-130 dependent manner. PLoS ONE. (2013) 8:e73266–13. 10.1371/journal.pone.0073266 24069182 PMC3772005

[131] Müller TA Grundler R Istvanffy R Rudelius M Hennighausen L Illert AL. Lineage-specific STAT5 target gene activation in hematopoietic progenitor cells predicts the FLT3(+)-mediated leukemic phenotype. Leukemia. (2016) 30:1725–33. 10.1038/leu.2016.72 27046463

[132] Schwaller J Parganas E Wang D Cain D Aster JC Williams IR. Stat5 is essential for the myelo- and lymphoproliferative disease induced by TEL/JAK2. Mol Cell. (2000) 6:693–704. 10.1016/S1097-2765(00)00067-8 11030348

[133] Hui W Cawston TE Richards CD Rowan AD. A model of inflammatory arthritis highlights a role for oncostatin M in pro-inflammatory cytokine-induced bone destruction via RANK/RANKL. Arthritis Res Ther. (2005) 7:R57–64. 10.1186/ar1460 15642143 PMC1064887

[134] Langdon C Kerr C Hassen M Hara T Arsenault AL Richards CD. Murine oncostatin M stimulates mouse synovial fibroblasts in vitro and induces inflammation and destruction in mouse joints *in vivo*. Am J Pathol. (2000) 157:1187–96. 10.1016/S0002-9440(10)64634-2 11021823 PMC1850181

[135] Hui W Rowan AD Richards CD Cawston TE. Oncostatin M in combination with tumor necrosis factor alpha induces cartilage damage and matrix metalloproteinase expression *in vitro* and *in vivo*. Arthritis Rheum. (2003) 48:3404–18. 10.1002/art.11333 14673992

[136] Plater-Zyberk C Buckton J Thompson S Spaull J Zanders E Papworth J. Amelioration of arthritis in two murine models using antibodies to oncostatin M. Arthritis Rheum. (2001) 44:2697–702. 10.1002/1529-0131(200111)44:11<2697::AID-ART450>3.0.CO;2-# 11710726

[137] Langdon C Leith J Smith F Richards CD. Oncostatin M stimulates monocyte chemoattractant protein-1- and interleukin-1-induced matrix metalloproteinase-1 production by human synovial fibroblasts *in vitro*. Arthritis Rheum. (1997) 40:2139–46. 10.1002/art.1780401207 9416850

[138] Fearon U Mullan R Markham T Connolly M Sullivan S Poole AR. Oncostatin M induces angiogenesis and cartilage degradation in rheumatoid arthritis synovial tissue and human cartilage cocultures. Arthritis Rheum. (2006) 54:3152–62. 10.1002/art.22161 17009243

[139] Hintzen C Quaiser S Pap T Heinrich PC Hermanns HM. Induction of CCL13 expression in synovial fibroblasts highlights a significant role of oncostatin M in rheumatoid arthritis. Arthritis Rheum. (2009) 60:1932–43. 10.1002/art.24602 19565514

[140] Cawston TE Curry VA Summers CA Clark IM Riley GP Life PF. The role of oncostatin M in animal and human connective tissue collagen turnover and its localization within the rheumatoid joint. Arthritis Rheum. (1998) 41:1760–71. 10.1002/1529-0131(199810)41:10<1760::AID-ART8>3.0.CO;2-M 9778217

[141] Richards CD Shoyab M Brown TJ Gauldie J. Selective regulation of metalloproteinase inhibitor (TIMP-1) by oncostatin M in fibroblasts in culture. J Immunol. (1993) 150:5596–603. 8515078

[142] Moran EM Mullan R McCormick J Connolly M Sullivan O FitzGerald O. Human rheumatoid arthritis tissue production of IL-17A drives matrix and cartilage degradation: synergy with tumour necrosis factor-alpha, Oncostatin M and response to biologic therapies. Arthritis Res Ther. (2009) 11:R113. 10.1186/ar2772 19627579 PMC2745795

[143] Duncan MR Hasan A Berman B. Oncostatin M stimulates collagen and glycosaminoglycan production by cultured normal dermal fibroblasts: insensitivity of sclerodermal and keloidal fibroblasts. J Investig Dermatol. (1995) 104:128–33. 10.1111/1523-1747.ep12613623 7798630

[144] Ihn H Tamaki K. Oncostatin M stimulates the growth of dermal fibroblasts via a mitogen-activated protein kinase-dependent pathway. J Immunol. (2000) 165:2149–55. 10.4049/jimmunol.165.4.2149 10925301

[145] Ihn H LeRoy EC Trojanowska M. Oncostatin M stimulates transcription of the human alpha2(I) collagen gene via the Sp1/Sp3-binding site. J Biol Chem. (1997) 272:24666–72. 10.1074/jbc.272.39.24666 9305936

[146] Hergovits S Mais C Haan C Costa-Pereira AP Hermanns HM. Oncostatin M induces RIG-I and MDA5 expression and enhances the double-stranded RNA response in fibroblasts. J Cell Mol Med. (2017) 21:3087–99. 10.1111/jcmm.13221 28560754 PMC5661242

[147] Boniface K Diveu C Morel F Pedretti N Froger J Ravon E. Oncostatin M secreted by skin infiltrating T lymphocytes is a potent keratinocyte activator involved in skin inflammation. J Immunol. (2007) 178:4615–22. 10.4049/jimmunol.178.7.4615 17372020

[148] Pohin M Guesdon W Mekouo AAT Rabeony H Paris I Atanassov H Oncostatin M overexpression induces skin inflammation but is not required in the mouse model of imiquimod-induced psoriasis-like inflammation. Eur J Immunol. (2016) 46:1737–51. 10.1002/eji.201546216 27122058

[149] Sanchez AL Langdon CM Akhtar M Lu J Richards CD Bercik P. Adenoviral transfer of the murine oncostatin M gene suppresses dextran-sodium sulfate-induced colitis. J Interferon Cytokine Res. (2003) 23:193–201. 10.1089/107999003765027393 12856331

[150] Mozaffarian A Brewer AW Trueblood ES Luzina IG Todd NW Atamas SP. Mechanisms of oncostatin M-induced pulmonary inflammation and fibrosis. J Immunol. (2008) 181:7243–53. 10.4049/jimmunol.181.10.7243 18981146

[151] Wong S Botelho FM Rodrigues RM Richards CD. Oncostatin M overexpression induces matrix deposition, STAT3 activation, and SMAD1 Dysregulation in lungs of fibrosis-resistant BALB/c mice. Lab Investig. (2014) 94:1003–16. 10.1038/labinvest.2014.81 24933422

[152] Langdon C Kerr C Tong L Richards CD. Oncostatin M regulates eotaxin expression in fibroblasts and eosinophilic inflammation in C57BL/6 mice. J Immunol. (2003) 170:548–55. 10.4049/jimmunol.170.1.548 12496442

[153] Ayaub EA Dubey A Imani J Botelho F Kolb MRJ Richards CD. Overexpression of OSM and IL-6 impacts the polarization of pro-fibrotic macrophages and the development of bleomycin-induced lung fibrosis. Sci Rep. (2017) 7:13281. 10.1038/s41598-017-13511-z 29038604 PMC5643520

[154] Miller M Beppu A Rosenthal P Pham A Das S Karta M. Fstl1 promotes asthmatic airway remodeling by inducing oncostatin M. J Immunol. (2015) 195:3546–56. 10.4049/jimmunol.1501105 26355153 PMC4811198

[155] Fritz DK Kerr C Tong L Smyth D Richards CD. Oncostatin-M up-regulates VCAM-1 and synergizes with IL-4 in eotaxin expression: involvement of STAT6. J Immunol. (2006) 176:4352–60. 10.4049/jimmunol.176.7.4352 16547273

[156] Machiyama T So T Okuyama Y Kobayashi S Phung HT Asao A. TNF receptor associated factor 5 controls oncostatin M-mediated lung inflammation. Biochem Biophys Res Commun. (2018) 499:544–550. 10.1016/j.bbrc.2018.03.186 29596835

[157] Mähler M Bristol IJ Leiter EH Workman AE Birkenmeier EH Elson CO. Differential susceptibility of inbred mouse strains to dextran sulfate sodium-induced colitis. Am J Physiol. (1998) 274:G544–51. 10.1152/ajpgi.1998.274.3.G544 9530156

[158] Beckwith J Cong Y Sundberg JP Elson CO Leiter EH. Cdcs1, a major colitogenic locus in mice, regulates innate and adaptive immune response to enteric bacterial antigens. Gastroenterology. (2005) 129:1473–84. 10.1053/j.gastro.2005.07.057 16285949

[159] Borm MEA He J Kelsall B Peña AS Strober W Bouma G. A major quantitative trait locus on mouse chromosome 3 is involved in disease susceptibility in different colitis models. Gastroenterology. (2005) 128:74–85. 10.1053/j.gastro.2004.10.044 15633125

[160] Farmer MA Sundberg JP Bristol IJ Churchill GA Li R Elson CO. A major quantitative trait locus on chromosome 3 controls colitis severity in IL-10-deficient mice. Proc Natl Acad Sci USA. (2001) 98:13820–5. 10.1073/pnas.241258698 11707574 PMC61125

[161] Buettner M Bleich A. Mapping colitis susceptibility in mouse models: distal chromosome 3 contains major loci related to Cdcs1. Physiol Genomics. (2013) 45:925–30. 10.1152/physiolgenomics.00084.2013 24022218

[162] Boulard O Kirchberger S Royston DJ Maloy KJ Powrie FM. Identification of a genetic locus controlling bacteria-driven colitis and associated cancer through effects on innate inflammation. J Exp Med. (2012) 209:1309–24. 10.1084/jem.20120239 22734048 PMC3405508

[163] Bleich A Büchler G Beckwith J Petell LM Affourtit JP King BL. Cdcs1 a major colitis susceptibility locus in mice; subcongenic analysis reveals genetic complexity. Inflamm Bowel Dis. (2010) 16:765–75. 10.1002/ibd.21146 19856416 PMC2857671

[164] Ermann J Garrett WS Kuchroo J Rourida K Glickman JN Bleich A. Severity of innate immune-mediated colitis is controlled by the cytokine deficiency-induced colitis susceptibility-1 (Cdcs1) locus. Proc Natl Acad Sci USA. (2011) 108:7137–41. 10.1073/pnas.1104234108 21482794 PMC3084042

[165] Ryzhakov G West NR Franchini F Clare S Ilott NE Sansom SN. Alpha kinase 1 controls intestinal inflammation by suppressing the IL-12/Th1 axis. Nat Commun. (2018) 9:3797. 10.1038/s41467-018-06085-5 30228258 PMC6143560

[166] Jostins L Ripke S Weersma RK Duerr RH McGovern DP Hui KY. Host-microbe interactions have shaped the genetic architecture of inflammatory bowel disease. Nature. (2012) 491:119–24. 10.1038/nature11582 23128233 PMC3491803

[167] Liu JZ van Sommeren S Huang H Ng SC Alberts R Takahashi A. Association analyses identify 38 susceptibility loci for inflammatory bowel disease and highlight shared genetic risk across populations. Nat Genet. (2015) 47:979–86. 10.1038/ng.3359 26192919 PMC4881818

[168] Gharavi AG Kiryluk K Choi M Li Y Hou P Xie J. Genome-wide association study identifies susceptibility loci for IgA nephropathy. Nat Genet. (2011) 43:321–7. 10.1038/ng.787 21399633 PMC3412515

[169] Cook S Ng B Dong J Viswanathan S DAgostino G Widjaja A IL-11 is a Therapeutic Target in Idiopathic Pulmonary Fibrosis. bioRxiv (2018). 10.1101/336537

[170] Lee CG Hartl D Matsuura H Dunlop FM Scotney PD Fabri LJ. Endogenous IL-11 signaling is essential in Th2- and IL-13–induced inflammation and mucus production. Am J Respir Cell Mol Biol. (2008) 39:739–46. 10.1165/rcmb.2008-0053OC 18617680 PMC2586049

[171] Tang W Geba GP Zheng T Ray P Homer RJ Kuhn C. Targeted expression of IL-11 in the murine airway causes lymphocytic inflammation, bronchial remodeling, and airways obstruction. J Clin Invest. (1996) 98:2845–53. 10.1172/JCI119113 8981933 PMC507752

[172] Waring PM Waring LJ Billington T Metcalf D. Leukemia inhibitory factor protects against experimental lethal Escherichia coli septic shock in mice. Proc Natl Acad Sci USA. (1995) 92:1337–41. 10.1073/pnas.92.5.1337 7877978 PMC42514

[173] Weber MA Schnyder-Candrian S Schnyder B Quesniaux V Poli V Stewart CL. Endogenous leukemia inhibitory factor attenuates endotoxin response. Lab Invest. (2005) 85:276–84. 10.1038/labinvest.3700216 15702085

[174] Trepicchio WL Bozza M Pedneault G Dorner AJ. Recombinant human IL-11 attenuates the inflammatory response through down-regulation of proinflammatory cytokine release and nitric oxide production. J Immunol. (1996) 157:3627–34. 8871663

[175] Maeshima K Takahashi T Nakahira K Shimizu H Fujii H Katayama H. A protective role of interleukin 11 on hepatic injury in acute endotoxemia. Shock. (2004) 21:134–8. 10.1097/01.shk.0000103386.98235.f6 14752286

[176] Bozza M Bliss JL Maylor R Erickson J Donnelly L Bouchard P. Interleukin-11 reduces T-cell-dependent experimental liver injury in mice. Hepatology. (1999) 30:1441–7. 10.1002/hep.510300616 10573523

[177] Shimizu T Shiratori K Sawada T Kobayashi M Hayashi N Saotome H. Recombinant human interleukin-11 decreases severity of acute necrotizing pancreatitis in mice. Pancreas. (2000) 21:134–40. 10.1097/00006676-200008000-00005 10975706

[178] Gao W Thompson L Zhou Q Putheti P Fahmy TM Strom TB. Treg versus Th17 lymphocyte lineages are cross-regulated by LIF versus IL-6. Cell Cycle. (2009) 8:1444–50. 10.4161/cc.8.9.8348 19342884 PMC2881570

[179] Cao W Yang Y Wang Z Liu A Fang L Wu F. Leukemia inhibitory factor inhibits T helper 17 cell differentiation and confers treatment effects of neural progenitor cell therapy in autoimmune disease. Immunity. (2011) 35:273–84. 10.1016/j.immuni.2011.06.011 21835648

[180] Curti A Ratta M Corinti S Girolomoni G Ricci F Tazzari P. Interleukin-11 induces Th2 polarization of human CD4(+) T cells. Blood. (2001) 97:2758–63. 10.1182/blood.V97.9.2758 11313268

[181] Bozza M Bliss JL Dorner AJ Trepicchio WL. Interleukin-11 modulates Th1/Th2 cytokine production from activated CD4+ T cells. J Interferon Cytokine Res. (2001) 21:21–30. 10.1089/107999001459123 11177577

[182] Hill GR Cooke KR Teshima T Crawford JM Keith JC Brinson YS. Interleukin-11 promotes T cell polarization and prevents acute graft-versus-host disease after allogeneic bone marrow transplantation. J Clin Invest. (1998) 102:115–23. 10.1172/JCI3132 9649564 PMC509072

[183] Chen Q Rabach L Noble P Zheng T Lee CG Homer RJ. IL-11 receptor alpha in the pathogenesis of IL-13-induced inflammation and remodeling. J Immunol. (2005) 174:2305–13. 10.4049/jimmunol.174.4.2305 15699166

[184] Ernst M Najdovska M Grail D Lundgren-May T Buchert M Tye H. STAT3 and STAT1 mediate IL-11-dependent and inflammation-associated gastric tumorigenesis in gp130 receptor mutant mice. J Clin Invest. (2008) 118:1727–38. 10.1172/JCI34944 18431520 PMC2323192

[185] Putoczki TL Thiem S Loving A Busuttil RA Wilson NJ Ziegler PK. Interleukin-11 is the dominant IL-6 family cytokine during gastrointestinal tumorigenesis and can be targeted therapeutically. Cancer Cell. (2013) 24:257–71. 10.1016/j.ccr.2013.06.017 23948300

[186] Johnstone CN Chand A Putoczki TL Ernst M. Emerging roles for IL-11 signaling in cancer development and progression: focus on breast cancer. Cytokine Growth Factor Rev. (2015) 26:489–98. 10.1016/j.cytogfr.2015.07.015 26209885

[187] West NR McCuaig S Franchini F Powrie F. Emerging cytokine networks in colorectal cancer. Nat Rev Immunol. (2015) 15:615–29. 10.1038/nri3896 26358393

[188] Juan TS-C Bolon B Lindberg RA Sun Y Van G Fletcher FA Mice overexpressing murine oncostatin M (OSM) exhibit changes in hematopoietic and other organs that are distinct from those of mice overexpressing human OSM or bovine OSM. Vet Pathol. (2009) 46:124–37. 10.1354/vp.46-1-124 19112126

[189] Clegg CH Rulffes JT Wallace PM Haugen HS. Regulation of an extrathymic T-cell development pathway by oncostatin M. Nature. (1996) 384:261–3. 10.1038/384261a0 8918875

[190] Boileau C Houde M Dulude G Clegg CH Perreault C. Regulation of extrathymic T cell development and turnover by oncostatin M. J Immunol. (2000) 164:5713–20. 10.4049/jimmunol.164.11.5713 10820248

[191] Shen MM Skoda RC Cardiff RD Campos-Torres J Leder P Ornitz DM. Expression of LIF in transgenic mice results in altered thymic epithelium and apparent interconversion of thymic and lymph node morphologies. EMBO J. (1994) 13:1375–85. 10.1002/j.1460-2075.1994.tb06391.x 8137821 PMC394955

[192] Louis I Dulude G Corneau S Brochu S Boileau C Meunier C. Changes in the lymph node microenvironment induced by oncostatin M. Blood. (2003) 102:1397–404. 10.1182/blood-2003-01-0316 12702501

[193] Metcalf D Gearing DP. A myelosclerotic syndrome in mice engrafted with cells producing high levels of leukemia inhibitory factor (LIF). Leukemia. (1989) 3:847–52. 2511382

[194] Malik N Haugen HS Modrell B Shoyab M Clegg CH. Developmental abnormalities in mice transgenic for bovine oncostatin M. Mol Cell Biol. (1995) 15:2349–58. 10.1128/MCB.15.5.2349 7739518 PMC230463

[195] Yoshida H Hunter CA. The immunobiology of interleukin-27. Annu Rev Immunol. (2015) 33:417–43. 10.1146/annurev-immunol-032414-112134 25861977

[196] Lucas S Ghilardi N Li J de Sauvage FJ. IL-27 regulates IL-12 responsiveness of naive CD4+ T cells through Stat1-dependent and -independent mechanisms. Proc Natl Acad Sci USA. (2003) 100:15047–52. 10.1073/pnas.2536517100 14657353 PMC299900

[197] Chen Q Ghilardi N Wang H Baker T Xie MH Gurney A. Development of Th1-type immune responses requires the type I cytokine receptor TCCR. Nature. (2000) 407:916–20. 10.1038/35038103 11057672

[198] Takeda A Hamano S Yamanaka A Hanada T Ishibashi T Mak TW. Cutting edge: role of IL-27/WSX-1 signaling for induction of T-bet through activation of STAT1 during initial Th1 commitment. J Immunol. (2003) 170:4886–90. 10.4049/jimmunol.170.10.4886 12734330

[199] Yoshida H Hamano S Senaldi G Covey T Faggioni R Mu S. WSX-1 is required for the initiation of Th1 responses and resistance to L. major infection. Immunity. (2001) 15:569–78. 10.1016/S1074-7613(01)00206-0 11672539

[200] Hall AO Beiting DP Tato C John B Oldenhove G Lombana CG. The cytokines interleukin 27 and interferon-γ promote distinct Treg cell populations required to limit infection-induced pathology. Immunity. (2012) 37:511–23. 10.1016/j.immuni.2012.06.014 22981537 PMC3477519

[201] Wang H Meng R Li Z Yang B Liu Y Huang F. IL-27 induces the differentiation of Tr1-like cells from human naive CD4+ T cells via the phosphorylation of STAT1 and STAT3. Immunol Lett. (2011) 136:21–8. 10.1016/j.imlet.2010.11.007 21115047

[202] Awasthi A Carrier Y Peron JPS Bettelli E Kamanaka M Flavell RA. A dominant function for interleukin 27 in generating interleukin 10-producing anti-inflammatory T cells. Nat Immunol. (2007) 8:1380–9. 10.1038/ni1541 17994022

[203] Murugaiyan G Mittal A Lopez-Diego R Maier LM Anderson DE Weiner HL. IL-27 is a key regulator of IL-10 and IL-17 production by human CD4+ T cells. J Immunol. (2009) 183:2435–43. 10.4049/jimmunol.0900568 19625647 PMC2904948

[204] Pot C Jin H Awasthi A Liu SM Lai C-Y Madan R. Cutting edge: IL-27 induces the transcription factor c-Maf, cytokine IL-21, and the costimulatory receptor ICOS that coordinately act together to promote differentiation of IL-10-producing Tr1 cells. J Immunol. (2009) 183:797–801. 10.4049/jimmunol.0901233 19570826 PMC2768608

[205] Romano M Sironi M Toniatti C Polentarutti N Fruscella P Ghezzi P. Role of IL-6 and its soluble receptor in induction of chemokines and leukocyte recruitment. Immunity. (1997) 6:315–25. 10.1016/S1074-7613(00)80334-9 9075932

[206] Watson C Whittaker S Smith N Vora AJ Dumonde DC Brown KA. IL-6 acts on endothelial cells to preferentially increase their adherence for lymphocytes. Clin Exp Immunol. (1996) 105:112–9. 10.1046/j.1365-2249.1996.d01-717.x 8697617 PMC2200481

[207] Kerfoot SM Raharjo E Ho M Kaur J Serirom S McCafferty DM. Exclusive neutrophil recruitment with oncostatin M in a human system. Am J Pathol. (2001) 159:1531–9. 10.1016/S0002-9440(10)62538-2 11583979 PMC1850489

[208] Yao L Pan J Setiadi H Patel KD McEver RP. Interleukin 4 or oncostatin M induces a prolonged increase in P-selectin mRNA and protein in human endothelial cells. J Exp Med. (1996) 184:81–92. 10.1084/jem.184.1.81 8691152 PMC2192668

[209] Barksby HE Hui W Wappler I Peters HH Milner JM Richards CD. Interleukin-1 in combination with oncostatin M up-regulates multiple genes in chondrocytes: implications for cartilage destruction and repair. Arthritis Rheum. (2006) 54:540–50. 10.1002/art.21574 16447230

[210] Kok S-H Hong C-Y Kuo MY-P Wang C-C Hou K-L Lin Y-T. Oncostatin M-induced CCL2 transcription in osteoblastic cells is mediated by multiple levels of STAT-1 and STAT-3 signaling: an implication for the pathogenesis of arthritis. Arthritis Rheum. (2009) 60:1451–62. 10.1002/art.24452 19404962

[211] Stawski L Trojanowska M Oncostatin M and its role in fibrosis. Connect Tissue Res. (2018) 19:1–10. 10.1080/03008207.2018.1500558 PMC6349219 30056769

[212] Nishimoto N Kanakura Y Aozasa K Johkoh T Nakamura M Nakano S. Humanized anti-interleukin-6 receptor antibody treatment of multicentric Castleman disease. Blood. (2005) 106:2627–32. 10.1182/blood-2004-12-4602 15998837

[213] Nishimoto N Yoshizaki K Miyasaka N Yamamoto K Kawai S Takeuchi T. Treatment of rheumatoid arthritis with humanized anti-interleukin-6 receptor antibody: a multicenter, double-blind, placebo-controlled trial. Arthritis Rheum. (2004) 50:1761–9. 10.1002/art.20303 15188351

[214] Villiger PM Adler S Kuchen S Wermelinger F Dan D Fiege V. Tocilizumab for induction and maintenance of remission in giant cell arteritis: a phase 2, randomised, double-blind, placebo-controlled trial. Lancet. (2016) 387:1921–7. 10.1016/S0140-6736(16)00560-2 26952547

[215] Bae S-C Lee YH. Comparison of the efficacy and tolerability of tocilizumab, sarilumab, and sirukumab in patients with active rheumatoid arthritis: a Bayesian network meta-analysis of randomized controlled trials. Clin Rheumatol. (2018) 37:1471–9. 10.1007/s10067-018-4006-5 29404725

[216] De Benedetti F Brunner HI Ruperto N Kenwright A Wright S Calvo I. Randomized trial of tocilizumab in systemic juvenile idiopathic arthritis. N Engl J Med. (2012) 367:2385–95. 10.1056/NEJMoa1112802 23252525

[217] Norelli M Camisa B Barbiera G Falcone L Purevdorj A Genua M. Monocyte-derived IL-1 and IL-6 are differentially required for cytokine-release syndrome and neurotoxicity due to CAR T cells. Nature. (2018) 24:739–48. 10.1038/s41591-018-0036-4 29808007

[218] Choy EH Bendit M McAleer D Liu F Feeney M Brett S. Safety, tolerability, pharmacokinetics and pharmacodynamics of an anti- oncostatin M monoclonal antibody in rheumatoid arthritis: results from phase II randomized, placebo-controlled trials. Arthritis Res Ther. (2013) 15:R132. 10.1186/ar4312 24286335 PMC3978888

[219] Moreland L Gugliotti R King K Chase W Weisman M Greco T. Results of a phase-I/II randomized, masked, placebo-controlled trial of recombinant human interleukin-11 (rhIL-11) in the treatment of subjects with active rheumatoid arthritis. Arthritis Res. (2001) 3:247–52. 10.1186/ar309 11438043 PMC34114

[220] Schwartz DM Kanno Y Villarino A Ward M Gadina M O'Shea JJ. JAK inhibition as a therapeutic strategy for immune and inflammatory diseases. Nat Rev Drug Discov. (2017) 16:843–62. 10.1038/nrd.2017.267 29104284

